# CD38 deficiency leads to a defective short-lived transcriptomic response to chronic graft-versus-host disease induction, involving purinergic signaling-related genes and distinct transcriptomic signatures associated with lupus

**DOI:** 10.3389/fimmu.2025.1441981

**Published:** 2025-02-10

**Authors:** Mercedes Zubiaur, Laura C. Terrón-Camero, Fernando Gordillo-González, Eduardo Andrés-León, Alicia Barroso-del Jesús, Luz María Canet-Antequera, María M. Pérez Sánchez-Cañete, África Martínez-Blanco, Marilú Domínguez-Pantoja, María Botia-Sánchez, Sonia Pérez-Cabrera, Nerea Bello-Iglesias, Antonio Alcina, Ana-Clara Abadía-Molina, Fuencisla Matesanz, Esther Zumaquero, Ramón Merino, Jaime Sancho

**Affiliations:** ^1^ Department of Cell Biology and Immunology, Institute of Parasitology and Biomedicine “López-Neyra” (IPBLN), Consejo Superior de Investigaciones Científicas (CSIC), Granada, Spain; ^2^ Bioinformatics Unit, IPBLN, CSIC, Granada, Spain; ^3^ Genomics, IPBLN, CSIC, Granada, Spain; ^4^ Flow Cytometry, IPBLN, CSIC, Granada, Spain; ^5^ Department of Biochemistry, Molecular Biology and Immunology III, School of Medicine, University of Granada (UGR), Granada, Spain; ^6^ Department of Microbiology, University of Alabama at Birmingham (UAB), Birmingham, AL, United States; ^7^ Department of Cell and Molecular Signaling, Institute of Biomedicine and Biotechnology of Cantabria (IBBTEC), University of Cantabria (UC) and CSIC, Santander, Spain

**Keywords:** CD38, purinergic-signaling, cGAS-STING, NLRP3-inflammasome, type I IFN signature, cGVHD lupus model, senescence, transcriptome signature

## Abstract

This study aimed to elucidate the transcriptomic signatures and dysregulated pathways associated with the autoimmune response in *Cd38^-/-^* mice compared to wild-type (WT) mice within the bm12 chronic graft-versus-host disease (cGVHD) lupus model. We conducted bulk RNA sequencing on peritoneal exudate cells (PECs) and spleen cells (SPC) at two and four weeks following adoptive cell transfer. We also analyzed cells from healthy, untreated mice. These analyses revealed a sustained upregulation of a transcriptional profile of purinergic receptors and ectonucleotidases in cGVHD WT PECs, which displayed a coordinated expression with several type I interferon-stimulated genes (ISGs) and with key molecules involved in the cyclic GMP-AMP synthase-stimulator of interferon genes (cGAS-STING) signaling pathway, two hallmarks in the lupus pathology. A second purinergic receptor transcriptomic profile, which included *P2rx7* and *P2rx4*, showed a coordinated gene expression of the components of the NLRP3 inflammasome with its potential activators. These processes were transcriptionally less active in cGVHD *Cd38^-/-^* PECs than in WT PECs. We have also shown evidence of a distinct enrichment in pathways signatures that define processes such as Ca^2+^ ion homeostasis, cell division, phagosome, autophagy, senescence, cytokine/cytokine receptor interactions, Th17 and Th1/Th2 cell differentiation in *Cd38^-/-^* versus WT samples, which reflected the milder inflammatory and autoimmune response elicited in *Cd38^-/-^* mice relative to WT counterparts in response to the allogeneic challenge. Last, we have shown an intense metabolic reprogramming toward oxidative phosphorylation in PECs and SPC from cGVHD WT mice, which may reflect an increased cellular demand for oxygen consumption, in contrast to PECs and SPC from cGVHD *Cd38^-/-^* mice, which showed a short-lived metabolic effect at the transcriptomic level. Overall, these findings support the pro-inflammatory and immunomodulatory role of CD38 during the development of the cGVHD-lupus disease.

## 1 Introduction

Systemic lupus erythematosus (SLE) is a female dominant autoimmune disease in which the autoreactive immune system causes inflammation and damage in multiple organs and tissues (1). CD38 expression on CD4^+^, CD8^+^, and CD25^+^ T cells was increased in SLE T cells and correlated with disease activity (2–5). Increased CD38 expression in T cells from patients with SLE may contribute to lupus pathogenesis because T cells produce Th1 and Th2 inflammatory cytokines when they are stimulated with CD38 antibodies (5). Increased CD38 expression greatly affects cellular metabolism by lowering intracellular NAD^+^ levels and decreasing NAD-dependent deacetylation performed by sirtuins (6). Thus, high levels of CD38 lead to decreased CD8 T-cell-mediated cytotoxicity and increased propensity to infections in patients with SLE (7). Moreover, CD38 reduces CD8^+^ T cell function by negatively affecting mitochondrial fitness (8). The relevance of these findings to lupus is that abnormal NAD-dependent deacetylation could be reverted pharmacologically or with anti-CD38 therapy (5, 9–11). Transcriptome analysis in a large number of SLE patients supported the prospect of CD38 inhibition for the treatment of SLE, corroborating the central role of plasma cells in SLE pathogenesis (12). In lupus models, this could also be approached by analyzing the functional effect of CD38 deficiency (13–16). Using the pristane lupus model, we have demonstrated the crucial role of CD38 in promoting aberrant inflammation and lupus-like autoimmunity *via* an apoptosis-driven mechanism, which requires TRPM2 expression (13). CD38 deficiency may improve lupus disease through an increase of IL-10-producing splenic regulatory B cells, which may cause a reduction of plasmacytoid dendritic cells and IFN-α production in the peritoneal cavity (14).

A chronic graft-*versus*-host reaction (cGVHD) induced in non-autoimmune C57BL/6 mice (B6) by the adoptive transfer of Ia-incompatible bm12 spleen cells results in a syndrome that closely resembles SLE in the spectrum of autoantibodies and immunopathology (17). In a previous paper we have shown that the absence of CD38 had a strong effect in the development of the bm12 cGVHD lupus model (18). Thus, there was a significant amelioration of the signs of the disease, including decreased expansion of T follicular helper (Tfh) cells, germinal center (GC) B cells, plasma cells, and Tbet^+^CD11c^hi^ B cells, while the expansion of regulatory T (Treg) cells and T follicular regulatory (Tfr) cells was normal, suggesting a defective response of *Cd38^-/-^* B cells to allogeneic help from CD38-suficient bm12 CD4^+^ T cells (18). Dysregulation of several cytokines and decreased protein abundance of STAT1 were also detected (18). Chronic autoimmune diseases such as SLE develop through positive feedback from inflammation. Among the cells involved in the inflammatory process, monocytes/macrophages could participate via a number of mechanisms. In this sense, CD38 expression in non-classical monocytes was recently linked to severe active SLE disease in a small group of patients (19). Moreover, it has been observed that lupus nephritis (LN) patients have an increased number of macrophages, whose CD38 expression is specifically activated and up-regulated, suggesting that CD38 might contribute to the regulation of LN development and progression by influencing immune responses (20). Therefore, it was of interest to analyze the transcriptomes of peritoneal exudate cells and spleen cells from cGVHD *Cd38^-/-^* mice vs WT mice to reveal the transcriptomic signatures and dysregulated pathways that are behind the distinct autoimmune response elicited in *Cd38^-/-^* mice vs WT mice (mild versus severe lupus disease). Transcriptomes of cGVHD affected mice along time were also compared with the transcriptomes of healthy controls to better understand the disease-state in a more dynamic approach, and to test whether in CD38-deficient mice there were signs of a faster resolution of the disease as suggested in our previous study. This approach has revealed a potential transcriptome signature in PECs that connects purinergic receptors with transcriptome signatures strongly associated with lupus such as the transcriptome signature of type I interferon-regulated genes, the cyclic GMP-AMP synthase-stimulator of interferon genes the cGAS-STING signaling pathway, and the NLRP3 inflammasome. This study also reveals a strong transcriptional reprogramming of metabolic pathways such as oxidative phosphorylation and thermogenesis in both PECs and spleen cells from cGVHD mice that may be related with the requirements for fast cell proliferation and cell differentiation in this lupus model.

## 2 Materials and methods

### 2.1 Mice

C57BL/6J (B6) (RRID: IMSR_JAX:000664) WT female mice were from Charles River. B6(C)-H2-Ab1bm12/KhEgJ (bm12) (RRID: IMSR_JAX:001162) female mice were from the Jackson Laboratory. B6.129P2-Cd38tm1Lnd/J (*Cd38^−/−^*) (RRID: IMSR_JAX:003727) female mice were backcrossed for 12 generations to the C57BL/6 J (B6) background and were provided by Dr. Frances Lund (UAB, Birmingham, USA). *Cd38^−/−^* mice were bred and maintained under specific pathogen-free conditions at the IPBLN-CSIC Animal Facility in Granada, Spain. The experimental procedures in animals at IPBLN-CSIC, Spain were approved by the Institutional Animal Care and Use Committee. The procedures follow the ARRIVE guidelines (21), and in accordance with the U.K. Animals (Scientific Procedures, Act, 1986) and associated guidelines (*EU Directive 2010/63/EU for animal experiments and RD53/2013*); and further revisions (*Commission Delegated Directive, EU 2024/1262*), and with the National Institutes of Health guide for the care and use of Laboratory animals (*NIH Publications No. 8023, revised 1978*).

### 2.2 The cGVHD inducible lupus model in C57BL/6 mice

In this model donor CD4^+^ T cells react to host B cells triggering the polyclonal activation of autoreactive B cells, and eventually, lupus-like syndrome (22). The procedure was described previously (18). We adapted the bm12 transfer model, as originally described by Morris et al. (23), and modified by Klarquist and Jansen (24). Eight to eighteen weeks-old B6 WT or *Cd38^−/−^* female mice were injected i.p. with 70-100 × 10^6^ isolated spleen cells from bm12 female mice as described (24). Both WT and *Cd38^-/-^* mice were adoptively transferred with bm12 CD38-sufficient spleen cells that contain an average of 20% of allo-reactive donor bm12 CD4^+^ T cells, which were fully functional (18, 24). Although experiments reported in the first description of the bm12 model found no significant difference in any disease parameter between male→male and female→female transfers (23), only female were used because SLE is a female dominant autoimmune disease (7, 25).

### 2.3 Spleen cells and peritoneal exudates cells

Spleen and PECs were harvested from mice after the sacrifice by inhalation of CO_2_, under sterile conditions, at 2 and 4 weeks post a chronic graft-versus-host reaction (cGVHD) induced in non-autoimmune C57BL6 (B6) female mice WT or *Cd38^-/-^*, by the adoptive transfer of Ia-incompatible bm12 spleen cells that results in a syndrome that closely resembles SLE in the spectrum of autoantibodies and immunopathology (17). PECs were obtained after i.p. injection of 5-6 ml of 1x Ca^2+^ and Mg^2+^ free PBS, pH 7.6, 0.5% BSA, 2 mM EDTA, 0.22 μm sterile filtered; followed by a gentle massage of the abdominal cavity. We collected peritoneal exudates (PE) containing PECs from the abdominal cavity by means of a small incision, with p1000-pipette sterile tips, into separate 15 ml sterile Falcon type tubes for each mouse. 15 ml-tubes were kept on ice, next they were spun down at 360 x g, 4˚C for 5 min, to isolate PECs in the cellular pellet. The spleens were obtained and SPC prepared as described (24). Cellular pellets containing either SPC or PECs were treated (1:2, v:v) with Ammonium Chloride Solution (STEMCELL Technologies, Ammonium Chloride 07850_07800), 5 min on ice; followed by 8.5 ml of DMEM complete medium, containing 5% heat inactivate Fetal Bovine Serum (FBS), pen: strep, 100 units/ml/10 microg/ml; 10 mM Hepes, 1X L glutamax, 2mM EDTA and 0.22 μm sterile filtered. SPC were spun down at 400 x g, 4˚C for 5 min, and PECs were spun down at 360 x g, 4˚C for 5 min. The supernatants were aspirated and a fresh DMEM complete medium was added to resuspend the cellular pellets. The experiments were repeated minimum three times. Three to 5 mice WT or *Cd38^-/-^* were included accordingly in each experiment. Isolated SPC and PECs were count and viability was analyzed on a hemocytometer with 1:1 Trypan blue solution (Sigma-Aldrich Cat# T8154). Spleen cells and PECs were washed thoroughly three times with cold 1x Ca^2+^ and Mg^2+^ free PBS, pH 7.6, 2 mM EDTA (filter sterilized in 0.22 mm filter); by centrifugation as indicated above (26).

### 2.4 Flow cytometry

Single cell suspensions of WT and *Cd38^-/-^* PECs were stained with the indicated anti-mouse antibodies (**Supplementary Table S1**) separated in different panels after blocking nonspecific binding with anti-mouse CD16/32 (BD Biosciences Cat# 553142, RRID: AB_394657), 1:200 dilution for 30-60 min, on ice, in staining media (1x PBS, w/o calcium or magnesium, 0.5% BSA, 2 mM EDTA, sterilized by means of 0.22 μm filter). Panel #2 (CD45R/B220-PE; CD11b-APC; CD4-BV480, and CD5-PerCP) was used to identify B1 cells, B2 cells, CD4^+^ T cells and macrophages. Panel #4 (F4/80-APC; CD11b-FITC, Ly6C-PE, and Ly6G-PerCP-Cy5.5) was used to identify large and small macrophages, Ly6C^hi^ monocytes and neutrophils. Each analyzed PEC sample corresponds to an individual mouse, either WT or *Cd38^-/-^*, from the corresponding experiment. Flow cytometry analyses at IPBLN-CSIC were performed as previously described (13). Ten thousand to 200.000 events per sample were acquired either in a FACSCalibur flow cytometer (BD Biosciences, RRID: SCR_000401), FACSaria III, or FACS Symphony (BD Biosciences) and analyzed with FlowJo software v10.10.0 (BD Biosciences) (FlowJo, RRID: SCR_008520). In each experiment, single cell suspensions of bm12 spleen cells were stained with panel #1 (TCR-β-FITC; CD4-PerCP-Cy5.5; CXCR5-biotin; Streptavidin-PE, and PD1-APC) to evaluate the numbers of allo-reactive donor bm12 CD4^+^ T cells that were adoptively to transferred to WT and *Cd38^-/-^* mice respectively, as previously described (18). Statistical analyses were performed using the GraphPad Prism 9 and 10.1.1 software (GraphPad Prism, https://www.graphpad.com/), using statistical tests as indicated in the text. Statistical significance was visualized as follows: ns = not significant (P > 0.05), * = P < 0.05, ** = P < 0.01, *** = P < 0.001, **** = P < 0.0001. All experiments have been done using three or more mice and representative images have been chosen for the figures.

### 2.5 RNA purification, library preparation and Illumina sequencing

RNA purification, library preparation and Illumina sequencing were carried out at the IPBLN Genomics Facility (CSIC, Granada, Spain). 5-10 x10^6^ washed and pelleted PECs and SPC, respectively, were lysed with RLT buffer (Qiagen, Cat number7926, Hilden, Germany) containing 1% beta-mercaptoethanol and following manufacturer guidelines. Cell lysates were immediately frozen in dry ice and kept at -80°C. Automated total RNA extraction was performed using RNeasy^®^ kit reagents and QIAcube^®^ robot (Qiagen). The RNA quality was evaluated by Bioanalyzer RNA 6000 Nano chip electrophoresis (Agilent Technologies) for a total of 62 RNA samples. Based on RNA quality and yield, 35 RNA samples were chosen, with RIN values of 10.51+/- 1.66 (mean +/- sem). Selected samples (**Supplementary Table S2**) included three biological replicates for every condition except for PECs from WT mice 4 weeks after the adoptive transfer of bm12 lymphocytes with two biological replicates. Each analyzed SPC sample corresponded to an individual mouse spleen, while each PEC sample was a pool of 2 or 3 mice from the corresponding experiment. RNA-seq libraries were prepared using Illumina mRNA stranded ligation kit (Illumina^®^) from 200 ng of input total RNA. Quality and size distribution of PCR-enriched libraries were validated through Bioanalyzer High Sensitivity DNA assay and concentration was measured on the Qubit^®^ fluorometer (Thermo). Final libraries were pooled in an equimolecular manner and then diluted and denatured as recommended by Illumina NextSeq^®^ 500 library preparation guide. The 72x2 nt paired-end sequencing was conducted on a NextSeq 500^®^ system with a final total output of 196 Gb and a quality score (Q30) of 91%.

### 2.6 Data analysis

The miARma-Seq pipeline, as detailed by Andres-Leon et al. (27, 28), was employed for the analysis of transcriptomic samples. This comprehensive workflow encompasses all stages from raw data processing to the calculation of differentially expressed genes (DEGs). Initially, the quality of raw data was assessed using FastQC software (29) to evaluate read quality. Following sample filtering, an average guanine-cytosine content of 51% and an average of 17M reads per sample were obtained, with no observed adapter accumulation or poor quality reads (q<30). Subsequently, Seqtk software (30) was utilized to standardize the number of reads per sample.

In the subsequent stage, miARma-Seq employed STAR (31) to align all sequences, resulting in 90.45% of properly aligned reads. For this purpose, the Mus musculus Gencode version M28 genome-build: mmGRCm28.p6 was utilized. Following alignment, featureCounts software (32) as utilized to map sequence reads to genes using reference gene annotation obtained from Gencode, based on the same assembly and genome build.

### 2.7 Differential expression

Differential expression analysis was conducted using the edgeR package (33). Genes that showed no expression (0 aligned reads across all samples) were removed, and the remaining genes were normalized using the trimmed mean of M-values (TMM) method (34). Additionally, reads per kilobase per million mapped reads (RPKM), counts per million (CPM), and log2-counts per million (log-CPM) were calculated for each gene in every sample (33). To assess the replicability of the samples, Principal Component Analysis (PCA) and Hierarchical Clustering of normalized samples were employed to provide a comprehensive overview of the similarity among RNA-sequencing samples (35, 36) (See **Supplementary Figure S1**). Differential expression analysis was performed between genes with a False Discovery Rate (FDR) value <0.05 were identified as DEGs. Additionally, the log_2_-fold change (log_2_FC) was utilized to assess the significance and magnitude of gene expression alterations between the two sample types. Several R packages are used to visualize our data effectively. Specifically, we used ‘ggplot2’ to create a Volcano plot in addition to the pheatmap and heatmaply packages taking advantage of their capabilities to generate heat maps with clustering settings. Data presented in the study are available at the Sequence Read Archive (SRA), BioProject PRJNA1118233.

### 2.8 Enrichment

Gene ontology (GO) enrichment analyses were evaluated for biological process ontology terms, molecular functions, and cellular components using the R clusterprofiler package (37). To identify the most relevant GO terms, the “Revigo” tool was used, using the R package rrvigo (38), which simplifies the visualization of functional enrichment results. For pathways analyses, Kyoto Encyclopedia of Genes and Genomes (KEGG; https://www.kegg.jp) canonical pathway database was used (39). To visualize shared and unique KEGG pathways for the different comparatives, Venn diagrams were generated using the online application Venny 2.1 (40). The heat-map displays the mean log_2_FC (logarithmic fold change) values calculated using edgeR, based on RNA-seq analysis of differential expression between experimental conditions log_2_FC values calculated using edgeR. The log_2_FC values were computed as the log_2_ ratio of normalized counts between the compared conditions. Each row represents a differentially expressed gene, while each column corresponds to one of the indicated pairwise comparisons performed. The values shown are the averages for each experimental condition (n is the number of biological replicates per condition, see **Supplementary Table S2**). Otherwise indicated, only genes with a log_2_FC greater than 2 or less than -2, in at least one comparison were represented in the heat-maps. The color scale reflects the magnitude and direction of gene expression changes: Red: Significant upregulation (positive log_2_FC values), Blue: Significant downregulation (negative log_2_FC values). Rows (genes) and columns (comparisons) are hierarchically clustered using Euclidean distance and the complete linkage method, grouping genes and conditions with similar expression patterns. This visualization highlights biologically meaningful changes associated with the specific GO or KEGG functional term under investigation.

### 2.9 *Cd38* gene expression

We found *Cd38* gene expression values in the count array of the Cd38-deficient samples, therefore, we decided to represent the alignment of the *Cd38* gene to check the coverage of these samples for this gene. We confirmed for CD38-deficient samples that no coverage was detected for exons 2 and 3 of the gene (**Supplementary Figure S2**), in comparison with the coverage of WT samples (**Supplementary Figure S2**, last track). As previously reported by other authors, these were the 2 exons deleted in the *Cd38^-/-^* mouse strain (41). Therefore, the expression of the *Cd38* gene in the CD38-deficient samples may come from the residual expression of the non-deleted exons of *Cd38* and, consequently, it does not lead to the functional production of the gene.

## 3 Results

### 3.1 Distinctive transcriptional profiling of cGVHD *Cd38^-/-^* PECs vs cGVHD WT PECs reveals significant differences in the cell subsets populating the peritoneal cavity of these mice

**Figure 1:**
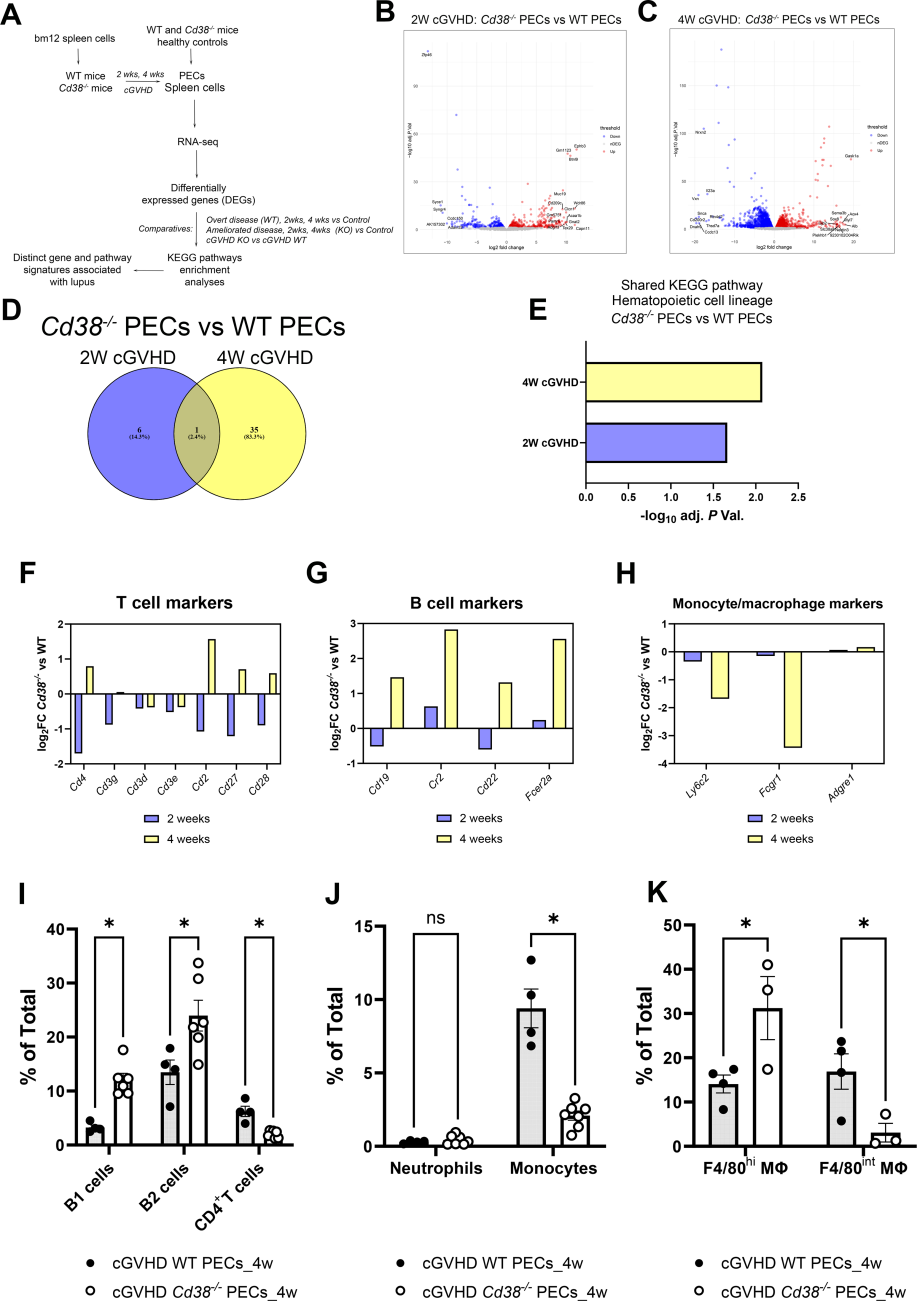
**(A)** Workflow of the experiments and analyses performed in this study. **(B)** Volcano plots showing upregulated DEGs (red dots) and downregulated DEGs (blue dots) of the 2-week comparative in PECs from cGVHD *Cd38^-/-^* vs cGVHD WT mice. **(C)** Volcano plot showing upregulated DEGs (red dots) and downregulated DEGs (blue dots) of the 4-week comparative in PECs from cGVHD *Cd38^-/-^* vs cGVHD WT mice. In both panels gene names are displayed for top DEGs. **(D)** Venn diagram showing the number of enriched KEGG pathways shared by the 2 comparatives, and those that were exclusively enriched at 2 weeks, or at 4 weeks of the allogeneic challenge. **(E)** Histograms represent the statistical significance of the hematopoietic cell lineage KEGG pathway shared by the 2 comparatives. **(F)** Log_2_ fold-changes in the expression of T cell markers genes in PECs at 2 weeks (violet bars) and 4 weeks (yellow bars) of the allogeneic challenge. **(G)** The same as in the **(F)** for B cell markers. **(H)** The same as in **(F, G)** for monocyte/macrophages markers. **(I)** Frequencies of B1 cells, B2 cells and CD4^+^ T cells in PECs from cGVHD WT mice and cGVHD *Cd38^-/-^* mice, 4 weeks after the allogeneic challenge. **(J)** Frequencies of neutrophils and pro-inflammatory Ly6C^hi^ monocytes in PECs of the same comparative as in **(I)**. **(K)** Frequencies of F4/80^high^ and F4/80^int^ macrophages in PECs of the same comparative as in **(I)**. Samples in **(I–K)** were analyzed by flow cytometry with specific markers as described in materials and methods. In **(I–K)**: ns, not significant (P > 0.05), * = P < from 0.01 to 0.05.

In a previous paper we have shown that in the absence of CD38 there was a significant amelioration of the signs of the disease in the cGVHD-lupus model, including decreased expansion of Tfh cells, GC B cells, plasma cells, and Tbet^+^CD11c^hi^ B cells, while the expansion of Treg cells and Tfr cells was normal, suggesting a defective response of *Cd38^-/-^* B cells to allogeneic help from bm12 CD4^+^ T cells (18). Therefore, it was of interest to test whether these features had a distinct gene expression profile. RNA sequencing was used to analyze the transcriptome of PECs and SPC from *Cd38^-/-^* mice and WT mice 2, and 4 weeks after the induction of the disease (see workflow in **Figure 1A**).

Two weeks after the adoptive transfer of bm12 into mice, 1727 DEGs were identified between *Cd38^-/-^* PECs and WT PECs (**Figure 1B**, **Supplementary Table S3**). Four weeks after the adoptive transfer, the number of DEGs increased up to 7301 (**Figure 1C**, **Supplementary Table S4**), indicating increased divergence in their transcription profile between *Cd38^-/-^* PECs and WT PECs. This increased divergence was even more evident when these DEGs were subjected to various enrichment analyses. Thus, there were 7 enriched KEGG pathways at 2 weeks, which increased up to 36 KEGG pathways at 4 weeks (**Supplementary Tables S5, S6**). Venn diagrams showed that only one pathway was shared between the 2 comparatives (**Figure 1D**). This was hematopoietic cell lineage (**Figure 1E**), which at 2 weeks showed decreased expression of genes such as *Cd4, Cd7, Cd5, Il5, Cd3g, Cd2, Cd36, and Cd44* in *Cd38^-/-^* vs WT PECs (**Supplementary Table S5**), many of them are T cells markers (**Figure 1F**). In this sense, decreased frequencies and numbers of CD11b^-^B220^-^CD4^+^ cells were observed by flow cytometry analysis (data not shown). To note is that, although not included in this KEGG pathway, we also observed significant decreased expression of *Cd28*, and *Cd27* genes in the 2-week comparative (**Figure 1F**). CD27 is expressed on both naïve and activated effector T cells, as well as on NK cells or activated B cells and plasma cells, and its expression is altered in SLE patients in various cell subsets (42–46). CD28 is one of the proteins expressed on T cells that provide co-stimulatory signals required for T cell activation and survival. In SLE patients lack of CD28 expression in specific T cell subsets may define their functional capabilities (47–50). Overall, these data may be indicative of decreased numbers of activated CD4^+^ T cells, and another unidentified CD4^+^ T cell subsets in the 2-week comparative.

In the 4-week comparative the hematopoietic cell lineage pathway showed 21 upregulated genes in *Cd38^-/-^* vs 17 in WT PECs, which made uncertain to predict any preponderance (**Supplementary Table S6**). A closer examination of the pathway indicated that gene markers of the T cell lineage, such as *Cd4*, *Cd2*, *Cd27*, *Cd28* (**Figure 1F**), and of the B cell lineage, such as *Cd19*, *Cr2*, *Cd22*, and *Fcer2* (**Figure 1G**) were upregulated in *Cd38^-/-^* PECs.

To better identify the major immune cell subsets from the lymphoid and myeloid lineages present in the peritoneal cavity after the allogeneic challenge, we used different multicolor flow cytometry antibody panels. We were able to identify the following subsets: B1 cells, B2 cells, macrophages (large and small), neutrophils, Ly6C^hi^ monocytes, Ly6C^lo^ monocytes, and CD4^+^ T cells (**Supplementary Figures S3**, **S4**).

In this sense, we observed by flow cytometry analysis increased proportions of B1 and B2 cells in *Cd38^-/-^* PECs vs WT PECs (**Figure 1I**, **Supplementary Figure S3**). In contrast, the proportion and numbers of CD4^+^ T cells were significantly lower in *Cd38^-/-^* PECs vs WT PECs (**Figure 1I**, **Supplementary Figure S3**, and data not shown), in apparent contradiction with the upregulated *Cd4* gene expression (**Figure 1F**). Therefore, *Cd4* gene expression in *Cd38^-/-^* PECs relative to WT PECs did not reflect its protein abundance or distribution within the CD4^+^ T cells detected by flow cytometry. We speculated that other cell subsets, such as a CD4^+^DEC-205^lo^CD11b^hi^ splenic DC subpopulation, which express high levels of CD4 RNA transcripts, and protein on its surface (51) might be present in PECs, although this required further investigation.

To note is that the proportion and numbers of pro-inflammatory Ly6C^hi^ monocytes were significantly lower in *Cd38^-/-^* PECs than in WT PECs 4 weeks after cGVHD induction (**Figure 1J**, **Supplementary Figure S4**, and data not shown), which was in agreement with the decreased expression of *Ly6c2*, the gene that encodes the Ly6C protein (**Figure 1H**). Other gene markers, which are predominant in monocytes/macrophages, and neutrophils, were downregulated (*Fcgr1*, *Tnf*, *Anpep*, *Il3ra*, *Il1r1*, *Il6ra*), or not differentially expressed (*Adgre1*) (**Figure 1H**, **Supplementary Table S6**). Although the proportion of macrophages in *Cd38^-/-^* PECs was not significantly different to that in WT PECs, we asked whether there were significant differences in the proportion of large peritoneal macrophages (LPMs), and small peritoneal macrophages (SPMs) (52). The LPM subset expresses high levels of the canonical macrophage surface markers, F4/80 and CD11b, while the SPM subset expresses substantially lower levels of CD11b and F4/80 (52). In our study, the proportion of F4/80^hi^ LPMs was significantly higher in *Cd38^-/-^* PECs than in WT PECs, while the proportion of F4/80^int^ SPMs was significantly lower (**Figure 1K**, **Supplementary Figure S4**). Since SPMs are originated from recruited pro-inflammatory Ly6C^hi^ monocytes to the peritoneal cavity during inflammatory conditions (52, 53), and both Ly6C^hi^ monocytes and SPMs are significantly augmented in the peritoneal cavity of WT mice relative to *Cd38^-/-^* mice 4 weeks after the cGVHD induction, our data are compatible with the induction of a stronger inflammatory response in the peritoneal cavity of WT mice than in *Cd38^-/-^* mice.

### 3.2 The transcriptional profile in cGVHD *Cd38^-/-^* PECs relative to cGVHD WT PECS revealed a decreased immune and inflammatory response at 4 weeks of the allogeneic challenge

**Figure 2:**
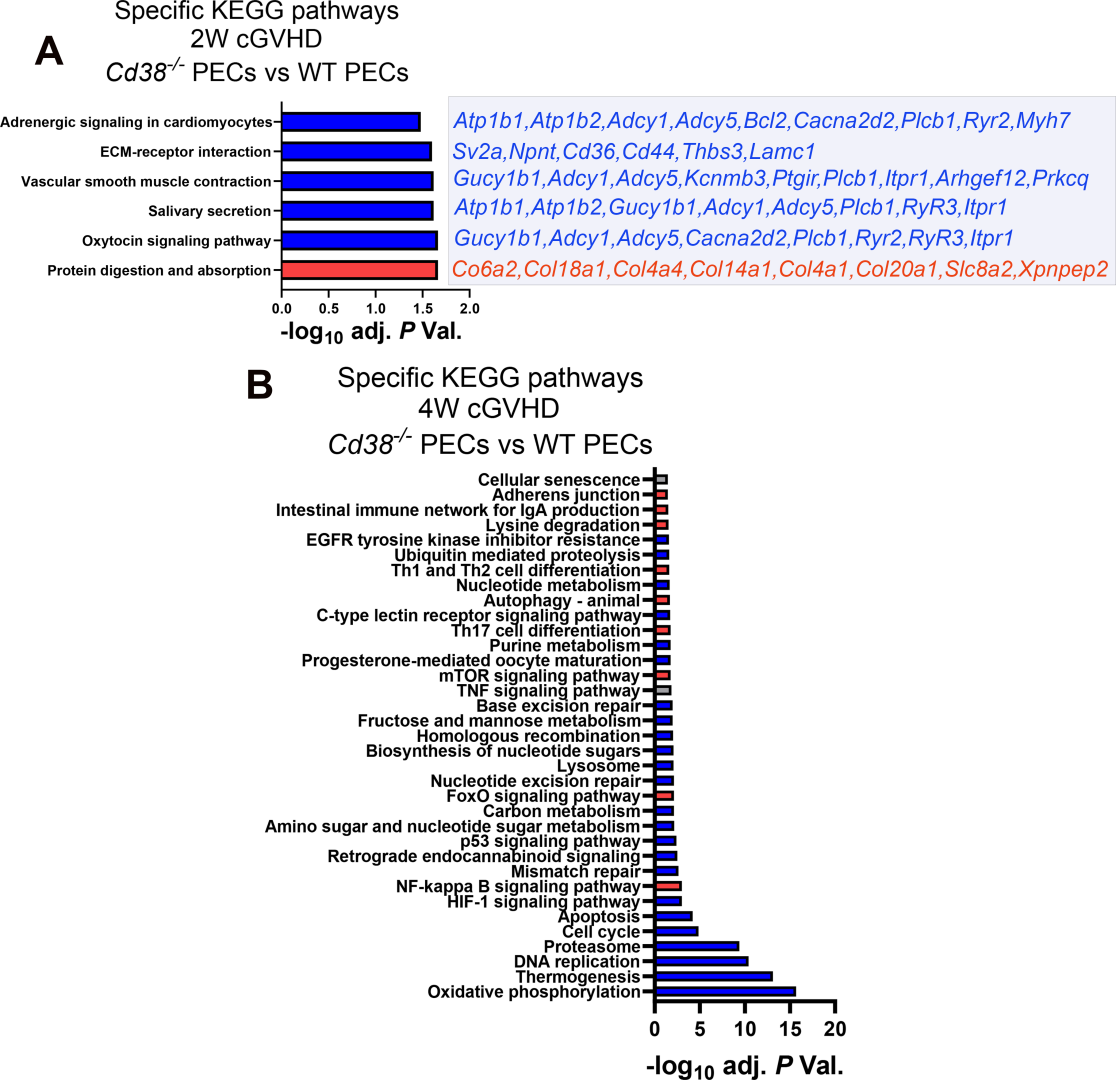
**(A)** Specific KEGG pathways of the 2-week comparative in PECs from cGVHD *Cd38^-/-^* vs cGVHD WT mice. Blue bars indicate pathways with ≥ 55% of downregulated genes. Red bars indicate pathways with ≥ 55% upregulated genes. Downregulated (blue) or upregulated (red) DEGs contributing to each pathway are listed on the right side of each bar. **(B)** Specific pathways of the 4-week comparative in PECs from cGVHD *Cd38^-/-^* vs cGVHD WT mice. In both panels blue bars indicate pathways with ≥ 55% of downregulated genes, and red bars indicate pathways with ≥ 55% upregulated genes. Grey bars in **(B)** indicate pathways with both upregulated and downregulated genes ≤ 54%, X axis represent the level of statistical significance (-log_10_ adjusted *P* value).

One of the KEGG pathways that was enriched in the 2-week comparative was the oxytocin signaling pathway (**Figure 2A**, **Supplementary Table S5**), where key elements were downregulated in *Cd38^-/-^* vs WT, including ryanodine receptors 2 and 3 (*Ryr2* and *Ryr3*), that together with decreased expression of *Cacna2d2*, *Plcb1* and *Itpr1*, are molecules involved in Ca^2+^ homeostasis and Ca^2+^-dependent signaling pathways (54–59). Some of the other enriched pathways such as salivary secretion, vascular smooth muscle contraction, and adrenergic signaling in cardiomyocytes shared many of these genes related with Ca^2+^-mediated signaling, either RyR-mediated or IP3-dependent mechanisms, or both (**Figure 2A**). In this sense, 4 out of the 8 GO BP enriched terms were related with calcium ion homeostasis and calcium-mediated signaling (data not shown). CD38 has been identified as an essential component for the rise in intracellular Ca^2+^ and oxytocin secretion (60, 61).

At 4 weeks, a larger number of genes related with oxidative phosphorylation, thermogenesis, DNA replication, proteasome, cell cycle, apoptosis, HIF-1, and p53 signaling pathways were downregulated in *Cd38^-/-^* relative to WT PECs, whereas a larger number of genes related with NF-κB, FOXO and mTOR signaling pathways were upregulated in *Cd38^-/-^* compared with WT PECs, together with genes involved in Th17 cell differentiation, autophagy, and Th1/Th2 cell differentiation (**Figure 2B**, **Supplementary Table S6**). Within the Th1/Th2 cell differentiation pathway, genes related with Th2 cell differentiation were upregulated in *Cd38^-/-^* PECs (*Jak1*, *Stat6*, *Gata3*), while Th1-related genes were downregulated (*Ifng*, *Ifngr2*, *Jak1/2*, *Stat1*), suggesting differential transcriptional regulation of these T cell subsets in *Cd38^-/-^* PECs vs WT PECs. Regarding Th17 cell differentiation, upregulated expression of *Rora* and *Rorc* genes was observed in *Cd38^-/-^* PECs relative to WT PECs. The proteins encoded by these genes, RORα and RORγt are essential for Th17 cell differentiation (62, 63). In contrast, *Il12rb1*, *Il23a*, *Foxp3*, *Stat1*, *Il27*, *Ifngr2*, *Ifng*, *Rxrg*, *Il21r*, and *Fos* genes, which are important for long-term Th17 cell survival and expansion (64–68), were downregulated in *Cd38^-/-^* PECs relative to WT PECs, which may be indicative of diminished survival capacity of *Cd38^-/-^* Th17-derived cells upon cGVHD induction.

Another interesting enriched KEGG pathway in the 4-week comparative was cellular senescence (**Figure 2B**). In *Cd38^-/-^* PECs, 30 out of 62 genes were upregulated and 32 genes were downregulated, which made difficult to assign this process to *Cd38^-/-^* or to WT (**Supplementary Table S6**). According with KEGG pathway database (https://www.kegg.jp/entry/mmu04218), in this pathway converged several signaling pathways. Therefore, it was of interest to search for differences in gene expression within each of these more specific pathways (**Supplementary Table S6**). Thus, genes associated to FOXO and mTOR signaling pathways were significantly upregulated in *Cd38^-/-^* PECs relative to WT PECs, while genes associated to calcium and p53 signaling pathways, cell cycle arrest, senescence-associated heterochromatin foci (SAHFs), and to senescence-associated secretory phenotype (SASP) were downregulated. The p53 signaling pathway elicits cell cycle arrest, and transcriptional activation of SAHF and SASP are key factors in senescence-associated inflammation (69, 70). Thus, significant decreased expression of *Il1a*, *Ccl*2, *Tnf*, and *Serpine1* genes, which are representative members of SASP components, was observed in *Cd38^-/-^* PECs relative to WT PECs (**Supplementary Tables S4**, **S6**). In contrast, *Tgfb2*, *Tgfbr2*, and *Cdkn2b* genes were upregulated. *Tgfb2* encodes TGF-β, and *Tgfbr2* encodes its receptor. TGF-β potently inhibits cell cycle progression at the G1 phase. *Cdkn2b* expression is induced by TGF-β, and encodes the cyclin-dependent kinase 4 inhibitor (p15) that is a potent inhibitor of cell cycle (71). In contrast, another inhibitor of cell cycle, *Cdkn1a*, which encodes p21, was downregulated. p21 is capable of inactivating all cyclin-dependent kinases (CDKs), thereby inhibiting cell cycle progression (72). To note is the downregulation of several cyclins, and CDKs, as well as the growth arrest and DNA damage inducible alpha (*Gadd45a*) and gamma *Gadd45g*), which are key elements in the control of cell cycle arrest via the p53-p21 pathway. Gene expression of other members of the p53-p21 pathway, such as *E2f*, *Mybl2*, *Foxm1*, and *Ln9* was downregulated, while *Rbl2*, which a potent inhibitor of E2F-trans-activation was upregulated (73). Likewise, increased *Sirt1* gene expression was detected in this 4-week comparative in *Cd38^-/-^* PECs, which is associated with protection from senescence via deactivation of p53 signaling (74). Therefore, the transcriptional data were compatible with an expected decreasing occurrence of cellular senescence in *Cd38^-/-^* PECs relative to WT PECs in cGVHD mice.

### 3.3 Differential gene expression of purinergic receptors, purinergic ectoenzymes, and signaling molecules involved in purinergic signaling pathways in *Cd38^-/-^* PECs relative to WT PECs after the cGVHD induction

**Figure 3:**
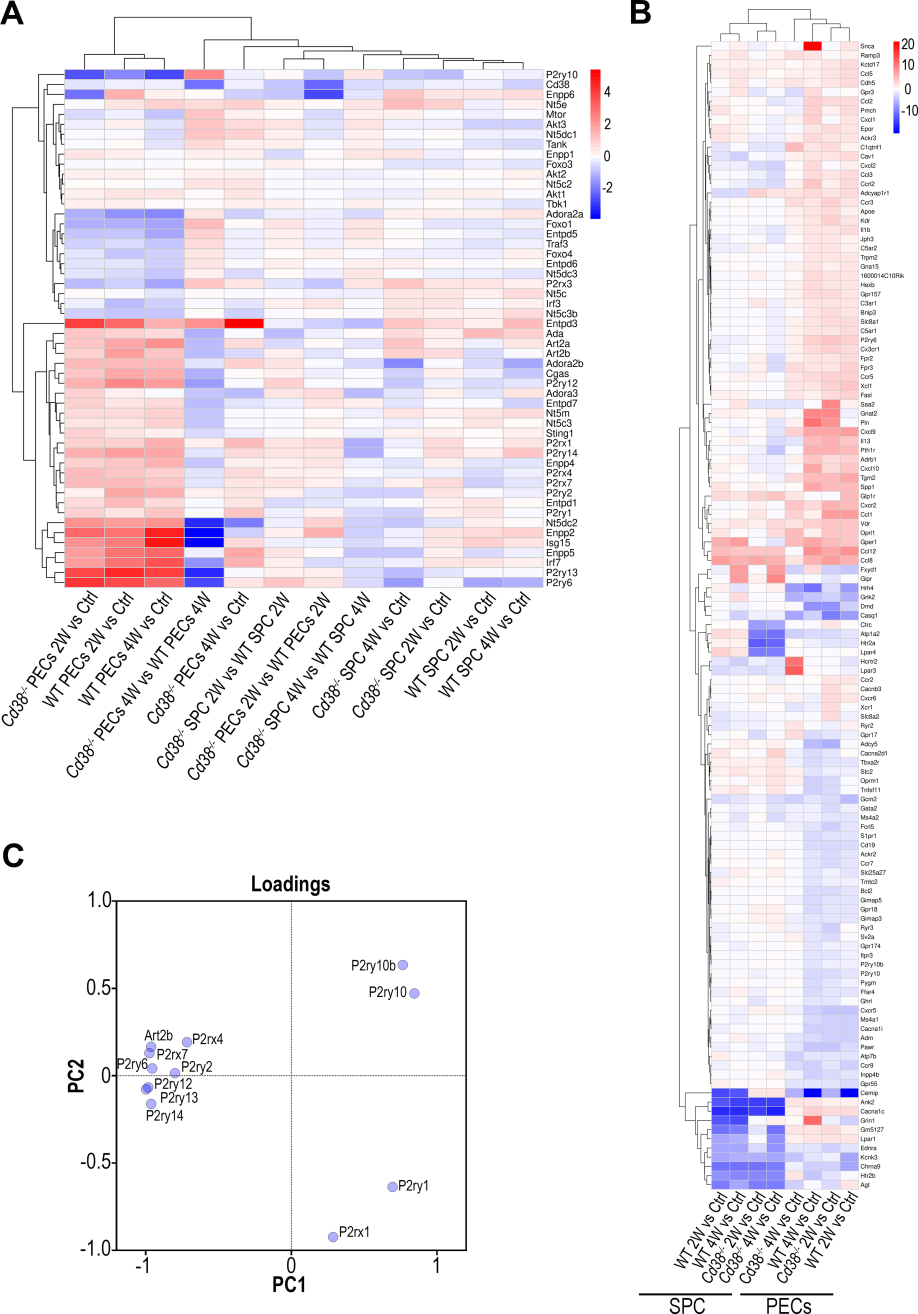
**(A)** Unsupervised hierarchical heat-map plot of the log_2_FC of the relative expression of the selected genes in the 12 comparatives indicated in the X-axis. **(B)** Unsupervised hierarchical heat-map plot of the log_2_FC of the relative expression of the genes associated with the GO term, Ca^2+^ ion homeostasis (GO:0055074), which was enriched in several comparatives. For this GO term, only DEGs with a log_2_FC ≥ 2, or log_2_FC ≤ -2 are represented, using as a reference the sample or samples where the GO term was enriched. The eight comparatives are indicated at the bottom. There was a perfect clustering of PECs samples separated from the spleen samples. **(C)** Loading plot of the 12 genes analyzed by PCA according with their differences in gene expression (log_2_FC, FDR ≤0.05, see **Table 1**) in the 4 comparatives tested (2-week and 4-week comparatives of cGVHD WT or cGVHD *Cd38^-/-^* PECs versus their respective controls from healthy untreated mice).

At 4 weeks of the cGVHD induction several metabolic pathways, including purine metabolism, and nucleotide metabolism appeared downregulated in *Cd38^-/-^* PECs relative to WT PECs (**Figure 2B**). Regarding the purine metabolism pathway, several genes encoding nucleotide-metabolizing ectoenzymes, which are involved in the extracellular metabolism of purines, were differentially expressed, including the ectonucleotide triphosphate diphosphohydrolase 5, *Entpd5*, which showed increased expression in this comparative, the ectonucleotide pyrophosphatase/phosphodiesterase family member 4, *Enpp4*, and adenosine deaminase, *Ada*, which both showed decreased expression (**Supplementary Table S6**). Since signaling through purinergic receptors is controlled by nucleotide-metabolizing ecto-enzymes, which regulate the availability of extracellular nucleotides, these findings prompted us to assess the differential expression of other genes related with purine metabolism, which are not included in the KEGG pathway, but are important key-players in inflammatory/anti-inflammatory processes (75). We focused our interest in the gene expression of P2X and P2Y purinergic receptors (*P2rx* and *P2ry*), adenosine receptors (*Adora*), ectoenzymes, including ecto-nucleoside triphosphate diphosphohydrolases (*Entpd*, CD39 family), 5’ ectonucleotidases (*Nt5*, CD73 family), ecto-nucleotide pyrophosphatase/phosphodiesterases (*Enpp*, CD203a family), ADP-ribosyltransferases (*Art2a* and *Art2b*), *Cd38*, and *Ada*. Last, we included in this search a number of genes encoding down-stream molecules which may be involved in purinergic receptor signaling. As shown in **Figure 3A**, unsupervised hierarchical heat-map representation of the log_2_FC of the selected genes in 12 comparatives showed a major cluster of 27 genes, which included 9 purinergic receptor genes such as *P2ry14*, *P2rx1*, *P2ry1*, *P2ry2*, *P2rx7*, *P2rx4*, *P2ry12*, *P2ry6*, and *P2ry13*; the ectonucleotidases *Entpd1*, *Entpd3*, *Enpp2*, *Enpp4*, *Enpp5*, *Entpd7*, *Enpp6*, and *Nt5e*; and other 5’-nucleotidases located in the cytosol or mitochondria such as *Nt5dc2*, *Nt5c3a*, and *Nt5m*; the adenosine receptors *Adora2b* and *Adora3*; the Adenosine deaminase, *Ada*; the T-cell ecto-ADP-ribosyltransferases *Art2a* and *Art2b*, and the signaling molecules, *Cgas*, *Sting1*, *Isg15* and *Irf7*, suggesting a similar pattern of expression. Moreover, this cluster showed upregulated expression in cGVHD PECs samples relative to Controls, in particular in WT PECs. In contrast, in SPC, this cluster of genes showed a more heterogeneous response, with less numbers of upregulated purinergic receptors, ectonucleotidases, and signaling molecules, but with increased expression of specific genes with clearly different kinetics than in PECs, which suggested a distinct pattern of expression.

To note is that increased *P2rx7* and *P2rx4* expression was clearly observed in most cGVHD PECs vs control comparatives, with a more sustained response in WT than in *Cd38^-/-^*. In contrast, in the spleen cells comparatives, *P2rx4* expression appeared downregulated, while *P2rx7* was upregulated, although to a lesser extent than in the *Cd38^-/-^* PECs 2W vs Control comparative (**Figure 3A**).

The transcriptional profile of this gene cluster may define a distinct transcriptional signature that connects purinergic signaling genes in combination with genes belonging to the so-called type I interferon transcriptome signature and genes of the cGAS-STING signaling pathway, which are key players in the SLE pathology (76). In summary, there were a number of purinergic receptors and other ectonucleotidases that participate in purinergic signaling, which were differentially expressed at transcriptional level in *Cd38^-/-^* PECs and spleen cells relative to their WT counterparts after cGVHD induction.

### 3.4 Differential gene expression of purinergic receptors, chemokine and chemokine receptors involved in Ca^2+^ homeostasis

It is known that purinergic signaling is connected to Ca^2+^ signaling. Thus, upon T cell receptor/CD3 stimulation, ATP release is increased and leads to autocrine activation of both P2X4 and P2X7 receptors, the amplification of the initial Ca^2+^ microdomain formation, and regulation of subsequent downstream responses (77, 78). Performing GO enrichment analyses of DEGs from cGVHD PECs vs Control PECs comparatives we noticed that many GO BP enriched terms were related with calcium ion homeostasis and calcium-mediated signaling. Therefore, we asked whether *P2rx4*, *P2rx7*, and other purinergic receptors genes were included within the gene set comprising the GO term, Ca^2+^ ion homeostasis (GO:0055074), which was enriched in several PECs comparatives. As shown in **Figure 3B** and **Supplementary Figure S5**, *P2rx7* and *P2rx4*, together with *P2rx1*, *P2ry6*, *P2ry10*, *P2ry10b*, *P2ry1* and *P2ry2* were selected. Only *P2ry10* appeared downregulated in both cGVHD *Cd38^-/-^* and cGVHD WT PECs relative to their respective healthy controls, while the others purinergic receptors genes showed upregulated expression, in particular *P2ry6*, and to a lesser extent *P2rx1*, *P2rx7*, *P2rx4*, *P2ry1*, and *P2ry2*. In PECs *P2ry6* formed part of a subcluster of strongly upregulated genes including a number of chemokines and chemokine receptors such as *Ccl1*, *Ccl12*, *Ccl8*, *Cxcl9*, *Cx3cr1*, and *Cxcr2* (**Figure 3B**). The heat-map representation showed that PECs samples were clearly segregated from spleen cells samples, and that there were several clusters of genes that seemed to have a coordinated expression as judged by their similar transcriptomic profiles. Within PECs, dendograms showed that the comparative of *Cd38^-/-^* PECs 4W relative to its control was more separated from the other 3 PECs samples, while in spleen cells the two *Cd38^-/-^* samples clustered separately from the two WT samples (**Figure 3B**).

### 3.5 Discriminative power of purinergic receptor gene expression

We observed that *P2rx1* and *P2ry1* genes exhibited a sustained increase in expression across the 4-time series samples when comparing the log_2_FC in gene expression values of cGVHD PECs to their respective healthy PECs controls (see **Table 1**). In contrast, the expression of the other 9 differentially expressed purinergic receptor genes declined exclusively in the 4-week comparison of cGVHD *Cd38^-/-^* PECs versus controls (see **Table 1**). To further identify the purinergic receptors genes that best discriminate between cGVHD WT mice and cGVHD *Cd38^-/-^* PECs, we employed a principal component analysis (PCA) multivariate statistical approach. We included the *Art2b* gene as a positive control, knowing the functional and physical interaction of ART2.2 and P2X7 in murine T cells (79). **Figure 3C** displays a loading plot showing the distribution of the 12 genes according with the variance in their relative expression profiles projected onto the first two principal components. Seven purinergic receptors - *P2rx4*, *P2rx7*, *P2ry2*, *P2ry6*, *P2ry12*, *P2ry13*, *P2ry14* -, and *Art2b* clustered in a relatively small area near the PC2 axis. Meanwhile, *P2ry10* and *P2ry10b* clustered in the upper right quadrant, and *P2rx1* and *P2ry1* were found in the lower right quadrant, close to the PC1 axis. This clustering confirmed and expanded upon the heat-map data (**Figure 3B**, **Supplementary Figure S5**), indicating that the tested genes were distributed in distinct areas corresponding to their differential expression in the four matched sets (2-week and 4-week comparative analyzed in WT and *Cd38^-/-^* mice). This pattern is consistent with a fundamentally different autoimmune and inflammatory response to the allogeneic challenge.

**Table 1:** Differential gene expression of selected 99purinergic receptors in PECs.

	P2rx1	P2rx4	P2rx7	P2ry1	P2ry2	P2ry6	P2ry10	P2ry10b	P2ry12	P2ry13	P2ry14	Art2b
2W vs Ctrl_WT	0.86	1.01	1.26	0.46	1.83	3.84	-1.76	-1.03	2.38	4.43	1.93	1.93
4W vs Ctrl_WT	1.61	0.85	1	1.01	1.48	3.11	-2.71	-2.2	2.03	3.89	1.86	1.03
2W vs Ctrl_*Cd38 ^-/-^*	0.98	1.31	1.23	0.91	**0.82**	4.27	-2.52	-1.53	1.63	3.72	1.48	0.92
4W vs Ctrl_*Cd38 ^-/-^*	1.25	0.64	**0.44**	1.1	**0.51**	**0.5**	**-0.19**	**0.097**	**-0.0003**	**-0.052**	0.57	-0.51

Numbers are the log_2_FC in gene expression in the cGVHD PECs vs Controls comparatives (**Supplementary Tables S7–10**).

In bold: not significant values.

### 3.6 The transcriptional profiles in cGVHD WT PECs along time revealed a strong and sustained immune and inflammatory response, in contrast to a short-lived transcriptional response in cGVHD *Cd38^-/-^* PECs

Transcriptomes of cGVHD affected mice along time were also compared with the transcriptomes of healthy controls to better understand the disease-state in a more dynamic approach, and to test whether in CD38-deficient mice there were signs of a faster resolution of the disease, as suggested in our previous study.

**Figure 4:**
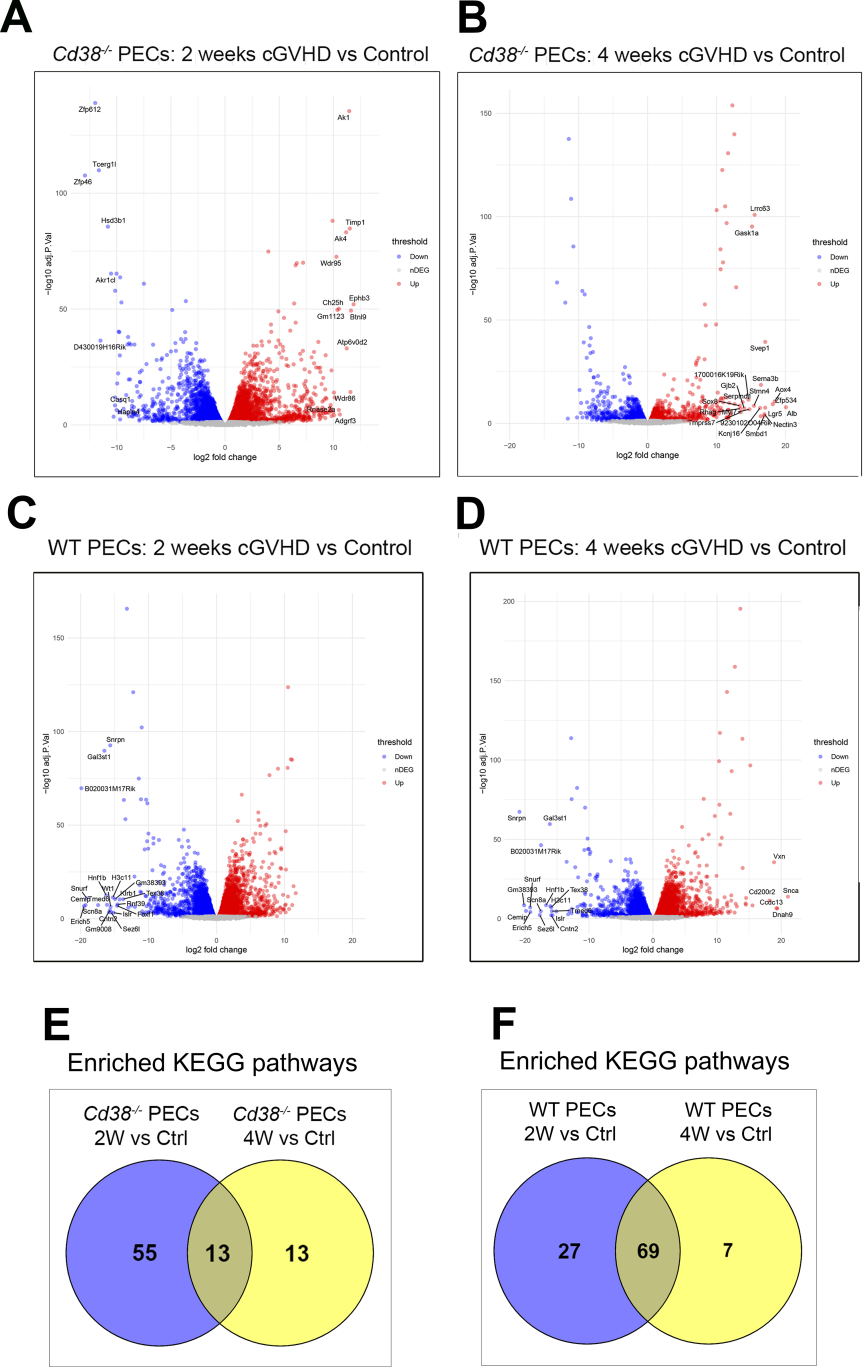
**(A)** Volcano plot showing upregulated DEGs (red dots) and downregulated DEGs (blue dots) in cGVHD *Cd38^-/-^* PECs 2 weeks after the adoptive transfer of bm12 cells vs Control *Cd38^-/-^* PECs from non-treated mice. **(B)** Volcano plot showing upregulated DEGs (red dots) and downregulated DEGs (blue dots) in cGVHD *Cd38^-/-^* PECs 4 weeks after the bm12 cells adoptive transfer vs Control *Cd38^-/-^* PECs from non-treated mice. **(C)** Volcano plot showing upregulated DEGs (red dots) and downregulated DEGs (blue dots) in WT PECs 2 weeks after the adoptive transfer of bm12 cells vs Control *Cd38^-/-^* PECs from non-treated mice. **(D)** Volcano plots showing upregulated DEGs (red dots) and downregulated DEGs (blue dots) in WT PECs 4 weeks after the adoptive transfer of bm12 cells vs Control *Cd38^-/-^* PECs from non-treated mice. **(E)** Venn diagram showing the number of shared and unique enriched KEGGs for the indicated comparatives. **(F)** Venn diagram showing the number of shared and unique enriched KEGGs for the indicated comparatives.

Volcano plots showed that 8562 genes were differentially expressed in *Cd38^-/-^* PECs 2 weeks after the adoptive transfer of bm12 spleen cells into the peritoneal cavity relative to control PECs from unchallenged mice (**Figure 4A**, **Supplementary Table S7**). The number of DEGs were reduced to 4157 4 weeks after the adoptive transfer (**Figure 4B**, **Supplementary Table S8**), suggesting a drastic reduction in the transcriptome response. In contrast, 9751 genes were differentially expressed in WT PECs at 2 weeks of the allogeneic challenge relative to controls (**Figure 4C**, **Supplementary Table S9**), and a similar number of genes, 9472, were differentially expressed at 4 weeks (**Figure 4D**, **Supplementary Table S10**), suggesting a similar transcriptome response.

To gain insight into the molecular pathways associated with the differences in autoimmune response between *Cd38^-/-^* and WT mice, enrichment analyses in KEGG pathways were performed in our comparative experiments along the time. In *Cd38^-/-^* PECs, 68 KEGG pathways were significantly enriched 2 weeks after the allogeneic challenge relative to controls (**Supplementary Table S11**). In contrast, only 26 KEGG pathways were enriched at 4 weeks (**Supplementary Table S12**), which was a drastic reduction relative to the 2 weeks-comparative. In WT PECs, at 2 weeks of the allogeneic challenge, 96 KEGG pathways were significantly enriched (**Supplementary Table S13**), and 76 at 4 weeks (**Supplementary Table S14**).

Venn diagrams showed that in *Cd38^-/-^* PECs only 13 enriched KEGG pathways were shared at both 2 and 4 weeks relative to controls (**Figure 4E**), while most enriched pathways belonged to the 2-week_vs_Ctrl comparative, where the bulk of the transcriptional response seemed to be concentrated. In contrast, Venn diagrams in WT PECs showed that 69 of these pathways were shared between both time points (**Figure 4F**), which suggested a more sustained transcriptional response than in *Cd38^-/-^* PECs.

**Figure 5:**
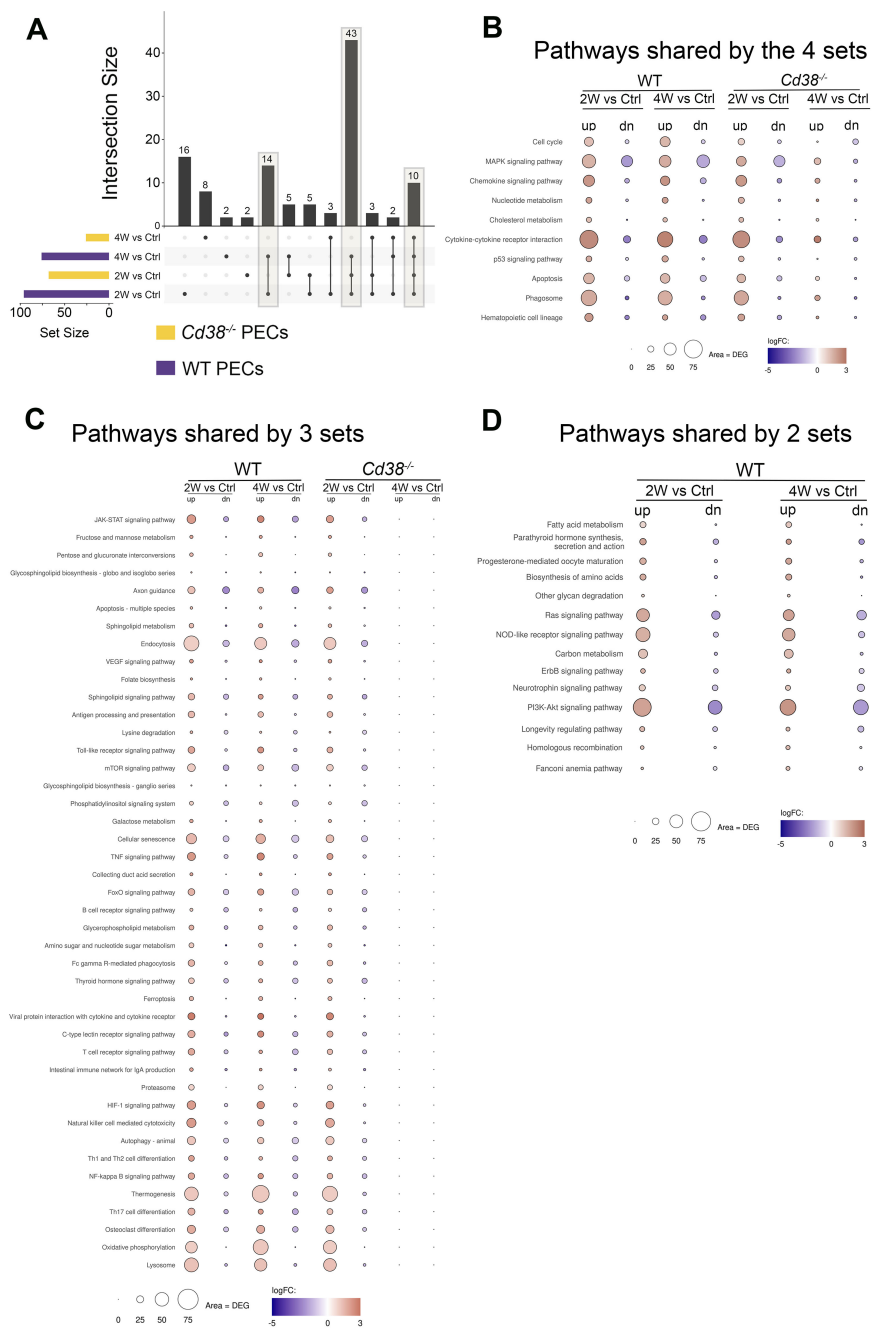
**(A)** The enriched KEGG pathways of these 4-time series or sets of PECs comparatives were matched and visualized using UpSet plots. Three different set intersections are highlighted. **(B)** The first highlighted intersection corresponded to the 10 pathways shared between all 4-time series. **(C)** The second intersection corresponded to the 43 pathways shared by *Cd38^-/-^* PECs 2W_vs_Ctrl, WT PECs 2W_vs_Ctrl, and WT PECs 4W_vs_Ctrl, but not with *Cd38^-/-^* PECs 4W_vs_Ctrl. **(D)** The third intersection corresponded to the 14 pathways shared by WT PECs 2W_vs_Ctrl and WT PECs 4W_vs_Ctrl. The mean log_2_FC of the upregulated DEGs (red circles) versus the mean log_2_FC of the downregulated DEGs (blue circles) per pathway were represented graphically. In this representation, the size of the circles was indicative of the number of upregulated or downregulated DEGs, and the intensity of the colors indicate the differences in log_2_FC.

To better understand the differences and similarities in the transcriptional response between *Cd38^-/-^* PECs and WT PECs, the enriched KEGG pathways of these 4-time series were matched and visualized using UpSet plots (80). In **Figure 5A**, three different set intersections are highlighted. The first highlighted intersection corresponded to the 10 pathways shared between all 4-time series. The second intersection corresponded to the 43 pathways shared by *Cd38^-/-^* PECs 2W_vs_Ctrl, WT PECs 2W_vs_Ctrl, and WT PECs 4W_vs_Ctrl, but not with *Cd38^-/-^* PECs 4W_vs_Ctrl. Finally, the third intersection corresponded to the 14 pathways shared by WT PECs 2W_vs_Ctrl and WT PECs 4W_vs_Ctrl.

**Figure 6:**
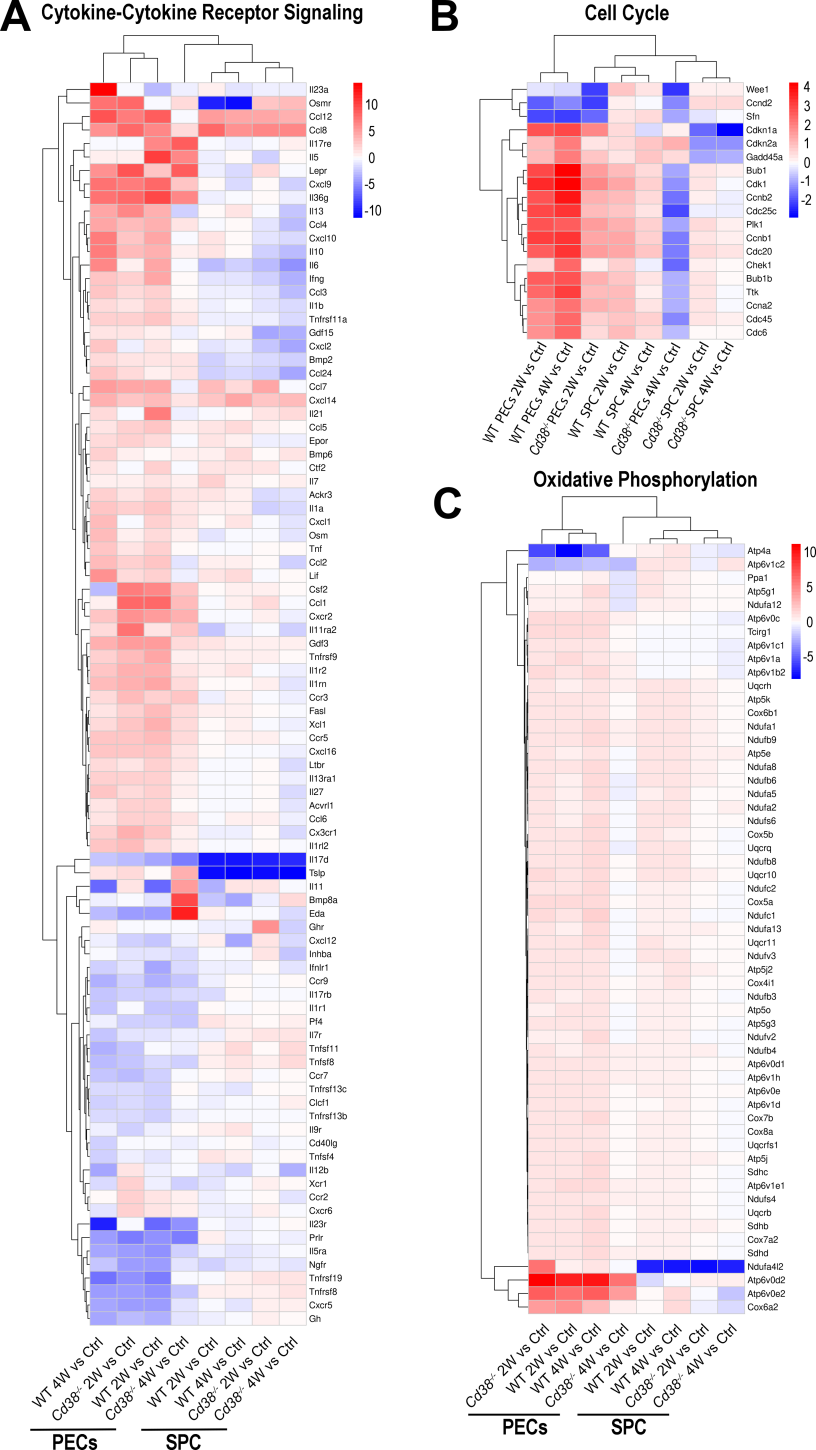
Unsupervised heat-map plot of the log_2_ fold changes of DEGs contributing to the indicated KEGG pathways: **(A)** Cytokine-Cytokine receptor signaling. **(B)** Cell Cycle process. **(C)** Oxidative phosphorylation. In panels **(A, B)**, only DEGs with a log_2_FC ≥ 2, or log_2_FC ≤ -2 are represented, using as a reference the sample or samples where the pathways were enriched. In panel **(C)**, only DEGs with a log_2_FC ≥ 1, or log_2_FC ≤ -1 are represented. The 8-time comparatives are indicated to the bottom of each panel. Vertical bars at the right of each panel represent the range of log_2_FC values. Red indicates upregulated gene expression. Blue indicates downregulated gene expression. Color intensity of the targeted genes indicates log_2_FC changes. The heat-maps of these three pathways, which include all DEGs, are shown in **Supplementary Figure S6A–C**.

To assess the intensity of the transcriptome response to the allogeneic challenge, the mean log_2_FC of the upregulated DEGs versus the mean log_2_FC of the downregulated DEGs per pathway were represented graphically. In this representation, the size of the circles was indicative of the number of upregulated or downregulated DEGs. From the 10 pathways shared between the 4 comparatives, cytokine-cytokine receptor interaction, phagosome, MAPK signaling pathway, chemokine signaling pathway, and apoptosis were the pathways with most upregulated DEGs involved, with a distinct pattern in *Cd38^-/-^* PECs (**Figure 5B**). Thus, in these comparatives, the number of upregulated DEGs corresponding to these pathways peaked at 2 weeks, declining sharply at 4 weeks. To note is that at 4 weeks the term cell cycle showed increased number of downregulated DEGs in *Cd38^-/-^* PECs, with no upregulated DEGs. This is better appreciated in a heat-map representation of the pathways cytokine-cytokine receptor interaction and cell cycle (**Figures 6A, B**, **Supplementary Figures S6A**, **S6B**). For these two pathways, dendograms showed that the *Cd38^-/-^* PECs_4W sample differed from the other three PECs samples, and it was closer to the gene profiling of spleen samples (**Figures 6A, B**, **Supplementary Figures S6A**, **S6B**).

In *Cd38^-/-^* PECs, differences in the kinetics of upregulated vs downregulated DEGs of the MAPK signaling pathway were observed with a peak of downregulated DEGs at 2 weeks. In contrast, in WT PECs, the maximum of downregulated DEGs associated with the MAPK signaling pathway occurred at 4 weeks. For most of the other pathways, including cell cycle, the number of upregulated DEGs remained stable at 2 and 4 weeks after the cGVHD induction, with a significant increase in the mean log_2_FC relative to that of the downregulated DEGs (**Figure 5B**). These findings were indicative of clear differences in the kinetics of activation of these pathways in *Cd38^-/-^* PECs versus WT PECs.

This distinctive gene profile was even more dramatically shown in 43 pathways that were significantly enriched in *Cd38^-/-^* PECs in the 2-week vs control comparative but not in the 4-week vs control, while in WT PECs the enrichments were observed in both comparatives (**Figure 5C**). The 10-top most significant pathways in *Cd38^-/-^* PECs 2-week vs control comparative were lysosome, oxidative phosphorylation, osteoclast differentiation, Th17 cell differentiation, thermogenesis, NF-κB signaling pathway, Th1 and Th2 cell differentiation, autophagy, NK cell mediated signaling pathway and HIF-1 signaling pathway. To note is that in WT PECs, for some pathways, such as oxidative phosphorylation and thermogenesis, the number of upregulated DEGs was higher at 4 weeks than at 2 weeks, resulting in enrichments with higher statistical significances (see **Figure 6C**, **Supplementary Figure S6C**, for a heat-map visualization of the changes in gene expression related with oxidative phosphorylation). Likewise, other pathways such as cellular senescence, TNF signaling pathway, and JAK-STAT signaling pathway, which are particularly interesting in the context of the lupus pathology, showed a stronger statistical significance in WT PECs than in *Cd38^-/-^* PECs in the 2-week vs control comparatives, and remained upregulated in the 4-week comparative in WT, and not in *Cd38^-/-^*.

We selected a number of known gene markers of cellular senescence, to evaluate more quantitatively the extent of this increased transcriptional activity related with this pathway (**Table 2**). Thus, in *Cd38^-/-^* PECs, *Cdkn1a*, *IL1a*, *Nampt*, *E2f1*, *Serpine1*, and to a lesser extent *Il6*, were significantly upregulated in the 2-week comparative and not in the 4-week comparative. In contrast, there was an upregulation of *Tgfb2* in the 4-week comparative and not in the 2-week comparative. Although, TGF-β plays an important role in senescence via inducing upregulation of *Cdkn2b* expression (71), it also a marker of the anti-inflammatory SASP, while IL-6, IL-1α, and IL-1β are markers of pro-inflammatory SASP (81, 82). As shown in **Table 2**, in WT PECs, the increased gene expression of selected senescence markers was quite stable with similar log_2_FC in the 2-week and 4-week comparatives, in contrast with the decline observed in the 4-week comparative of *Cd38^-/-^* PECs. To note is the significant increased expression of *Il6*, and decreased expression of *Sirt1*, a putative protector of senescence (74). The relative increase in *Tgfb2* expression at 4 weeks did not reach statistical significance (FDR = 0.09417).

**Table 2:** Differential gene expression of selected senescence markers.

	*Cdkn1a*	*IL1a*	*IL6*	*Tgfb2*	*Nampt*	*Sirt1*	*E2f1*	*Serpine1*
2W vs Ctrl_WT	2.69	2.80	4.44	0.04	1.93	-0.84	1.11	7.79
4W vs Ctrl_WT	2.96	3.08	6.07	**0.69**	1.89	-1.25	1.74	7.90
2W vs Ctrl_*Cd38 ^-/-^*	1.85	2.26	0.36	-0.07	1.36	-0.71	0.91	6.62
4W vs Ctrl_*Cd38 ^-/-^*	**0.12**	1.37	**-0.03**	1.64	**0.03**	**-0.16**	-0.51	**0.01**

Numbers are the log_2_FC in gene expression in cGVHD PECs vs Controls comparatives (**Supplementary Tables S7–10**).

In bold: not significant values.

### 3.7 Distinct upregulation of genes involved in NOD-like receptor signaling in *Cd38^-/-^* PECs vs WT PECs during cGVHD induction

**Figure 7:**
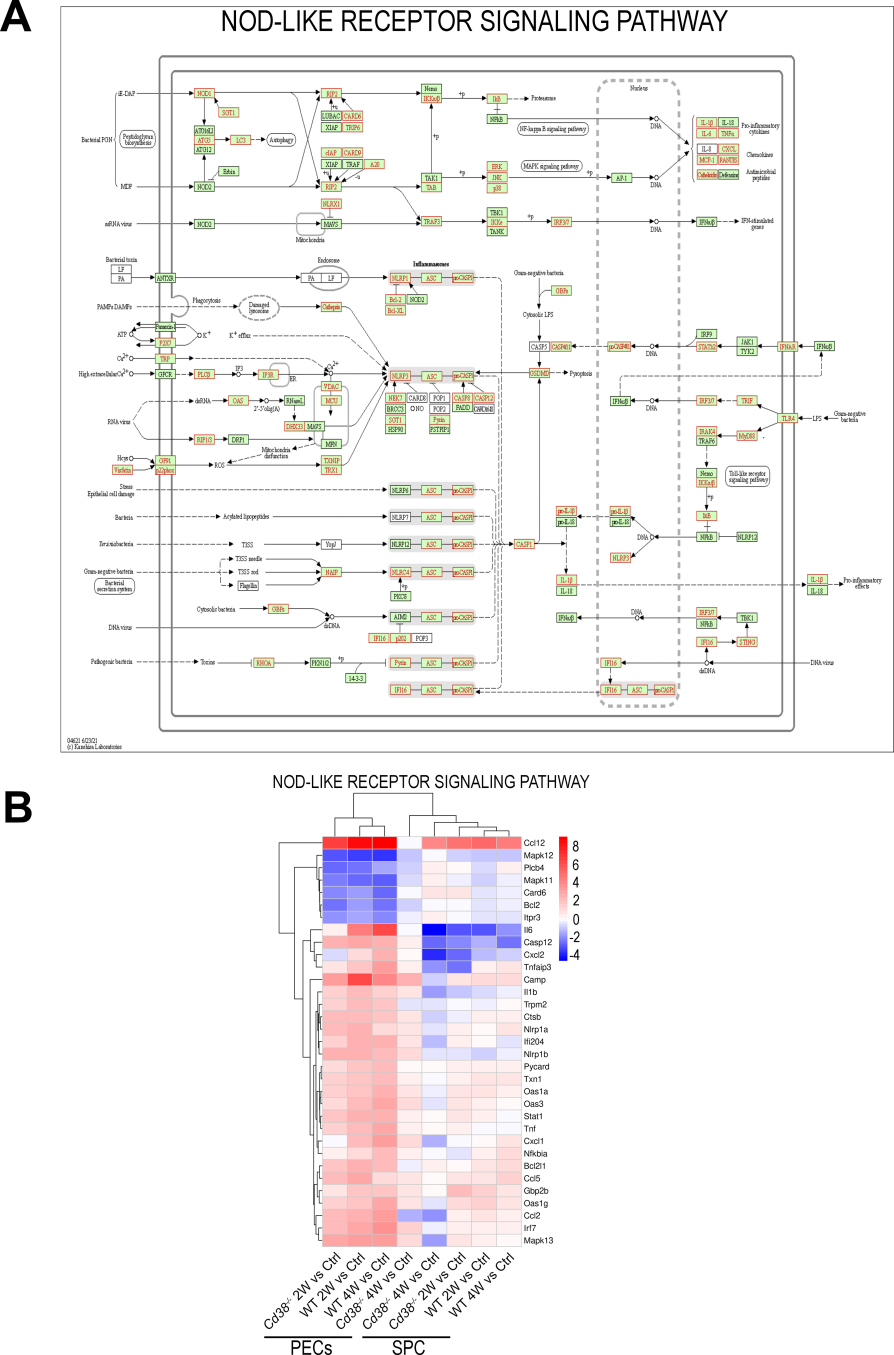
**(A)** NOD-like receptor signaling pathway reference KEGG map 04621 highlighting (in red) DEGs detected in the cGVHD WT PECs 2W vs Ctrl WT PECs comparative. Similar results were obtained in the cGVHD WT PECs 4W vs Ctrl WT PECs comparative, but not in the respective *Cd38^-/-^* comparatives (data not shown). **(B)** Unsupervised heat-map plot of the log_2_ fold changes of DEGs contributing to NOD-like receptor signaling. DE gene names are shown to the right of each heat-map panel. Only DEGs with a log_2_FC ≥ 2, or log_2_FC ≤ -2 are represented, using as a reference the sample or samples where the KEGG pathway was enriched. The 8-time comparatives are indicated to the bottom of each panel. Vertical bar at the right of the panel represents the range of log_2_FC values. Red indicates upregulated gene expression. Blue indicates downregulated gene expression. Color intensity of the targeted genes indicates log_2_FC changes. The heat-map of this pathway, which includes all DEGs, is shown in **Supplementary Figure S6D**. Permission to use the KEGG map04621 has been granted by Kanehisa laboratories.

To note is that 14 pathways were exclusively enriched in WT PECs and not in *Cd38^-/-^* PECs, including Ras signaling pathway, NOD-like receptor signaling pathway, fatty acid metabolism and the PI3K-Akt signaling pathway (**Figure 5D**). Regarding the NOD-like receptor (NLR) signaling pathway, there is a connection of cytosolic NLRs with inflammatory and autoimmune disorders, including lupus (83, 84). These innate immune pattern recognition molecules are essential for controlling inflammatory mechanisms through induction of cytokines, chemokines, and anti-microbial genes (84, 85). As shown in **Figure 7A**, and **Supplementary Figure S6D**, 2 weeks and 4 weeks after cGVHD induction in WT PECs there was increased expression of the NLR-encoding genes: *Nlrp3*, *Nod1* (*Nlrc1*), *Nlrp1a*, *Nlrp1b*, *Nlrx1*, *Nlrc3*, and *Ifi204* in comparison to control non-treated WT PECs (see also **Supplementary Tables S9**, **S10**). In contrast, *Nlrc4* expression was downregulated during cGVHD induction, while *Mefv*, which encodes the protein Pyrin, showed increased expression only at 2 weeks (**Supplementary Figure S6D**). All these NLRs are the cytosolic sensor components of distinct inflammasome complexes, which are assembled with the proteins encoded by *Pycard* and *Casp1*, and that were overexpressed in WT PECs after cGVHD induction (**Figure 7A**, **Supplementary Figure S6D**). Inflammasomes activate Caspase-1, which in turn cleaves pro-IL1β and pro-IL-18, allowing their secretion as mature IL-1β and IL-18. *Il1b* was also overexpressed in WT PECs upon the allogeneic challenge. Moreover, *P2rx7*, *Trpm2*, and *Tlr4* genes were upregulated, which are genes encoding potential activators of the NLRP3-inflammasome complex (86, 87).

To note is the increased and stable expression of *Nampt* in WT PECs after cGVHD induction, while in *Cd38^-/-^* PECs occurred only in the 2-week vs Control comparative (**Supplementary Figure S6D**, bottom). NAMPT is a key enzyme in the NAD^+^ biosynthesis, and extracellular NAMPT acts as a cytokine that modulates the immune and inflammatory response (88). Moreover, inhibition of NAMPT activity by FK866 has been shown to decrease inflammatory cytokine release, which was mechanistically linked with altered monocyte/macrophage biology and skewed macrophage polarization, by reducing CD86, CD38, MHC-II and IL-6 expression and promoting CD206, Egr2 and IL-10, which can be used to attenuate acute intestinal inflammation (89).

In WT PECs other nucleotide sensors were upregulated, including *Cgas* and *Sting1*, main components of the cGAS-STING signaling pathway, and a number of downstream genes associated with the *in vivo* type I IFN response such as *Ifnar2*, *Stat1*, *Stat2*, *Irf7*, *Isg15*, *Mx1*, *Oas1a*, *Oas1g*, *Oas2*, and *Oas3*. Most of these ISGs showed gene expression changes of more than two-fold (**Figures 3A**, **7B**).

*Nlrp1a*, *Nlrp1b*, and *Ifi204* showed the highest increased expression among the NLR-encoding genes (log_2_FC > 2, **Figure 7B**). *Ifi204* encodes the interferon-γ-inducible protein (Ifi-204), the murine orthologous gene of human IFI16. As shown in **Figure 7A**, IFI16/Ifi-204 may act at different levels within the NOD-like receptor signaling pathway. IFI16 is known as a cytosolic DNA sensor, which forms an inflammasome in response to herpesviruses, lentiviruses and intracellular DNA, leading to caspase-1 activation and pyroptosis (90–92). Moreover, IFI16 and Ifi-204 proteins mediate type I IFN induction via recruiting STING (90, 91).

In contrast, another DNA sensor, *Tlr9*, that also induces type I IFN responses, was downregulated in both WT and *Cd38^-/-^* PECs (**Supplementary Figure S6D**, **Figure 7B**). In this sense, SLE B cells show higher expression of interferon regulated genes and nucleic acid sensors than healthy B cells (76).

Pro-inflammatory cytokines such as *Il6*, and *Tnf*; and chemokines such as *Ccl2*, *Ccl5*, *Ccl12*, *Cxcl1*, and *Cxcl2*, which have chemotactic activity for monocytes, neutrophils and other cell-types, and are expressed at sites of inflammation, also showed sustained upregulated gene expression in WT PECs in response to the allogeneic challenge, with log_2_FC > 2 (**Figure 7B**, **Supplementary Tables S9**, **S10**).

### 3.8 Extensive downregulation of key immune response signaling pathways in *Cd38^-/-^* spleen cells upon cGVHD induction

The above results strongly suggested that *Cd38^-/-^* PECs responded to the adoptive transfer of bm12 cells with a peak of transcriptional activity at 2 weeks, and a sharp decline thereafter, while in WT PECs the response was more intense and long-lasting. It was of interest to test whether this phenomenon also occurred in a secondary lymphoid organ such as the spleen where the allogeneic immune response is generated, and altered metabolic pathways are likely to occur (93).

**Figure 8:**
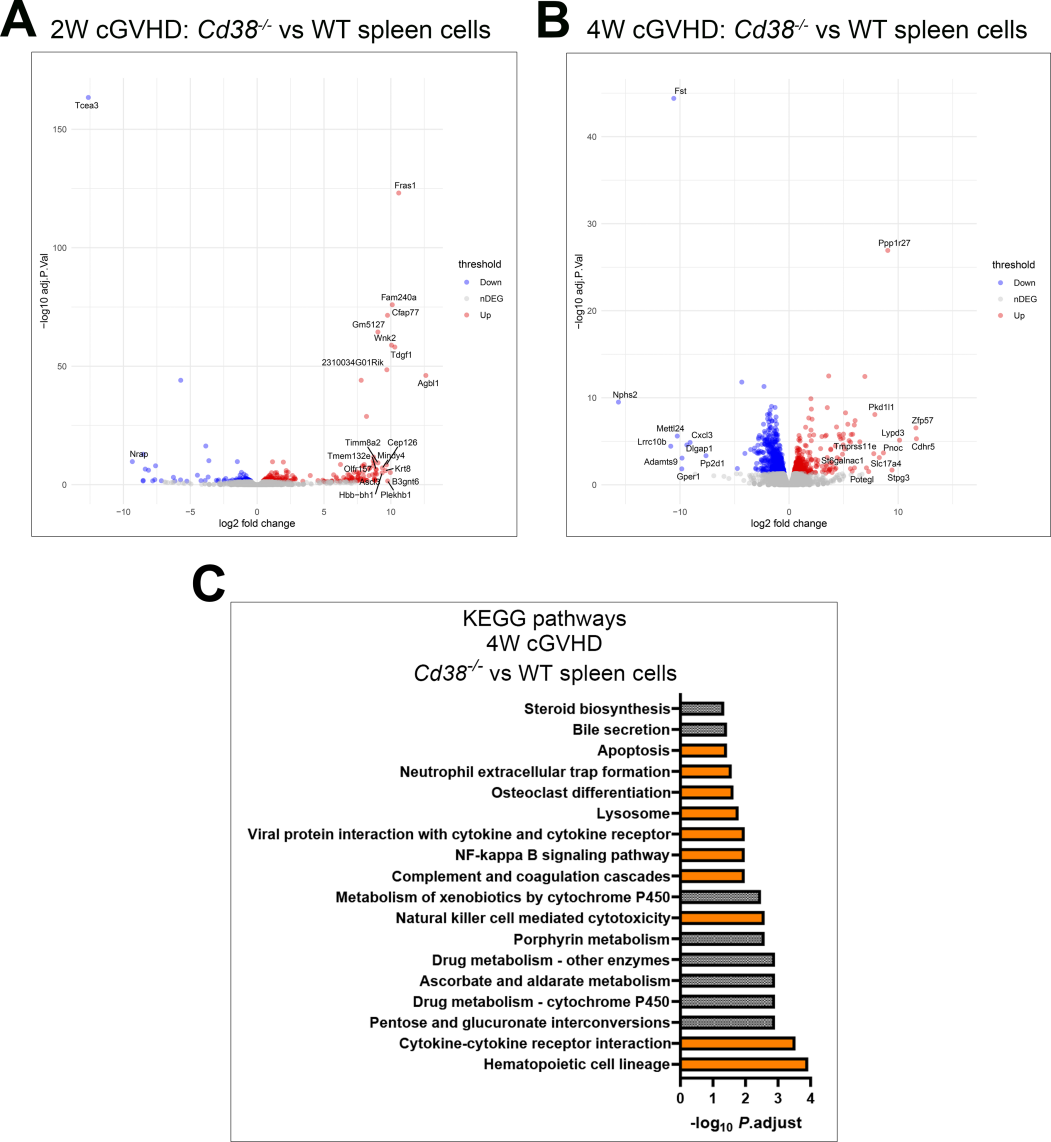
**(A)** Volcano plot of the DEGs upregulated (red circles), and downregulated (blue circles) in *Cd38^-/-^* vs WT spleen cells 2 weeks after the bm12 cell transfer. **(B)** Volcano plots of the DEGs upregulated (red circles), and downregulated (blue circles) in *Cd38^-/-^* vs WT spleen cells 4 weeks after the bm12 cell transfer. **(C)** KEGG pathways enriched in the 4-week comparative. Histograms represent the level of significance as ‐log_10_ of the P adjusted value. Pathways related with immune response are highlighted in orange.

First, we asked whether there were differences in gene expression in *Cd38^-/-^* spleen cells relative to WT spleen cells 2 weeks and 4 weeks upon the adoptive transfer of bm12 cells. 867 DEGs were identified at 2 weeks (**Figure 8A**, **Supplementary Table S15**), and 1410 DEGs at 4 weeks after the adoptive transfer (**Figure 8B**, **Supplementary Table S16**). At 2 weeks there were not enriched KEGG pathways, while at 4 weeks 10 out of the 18 enriched KEGG pathways were related with immune or inflammatory responses (**Supplementary Table S17**, **Figure 8C**). To note is that *Siglech*, which is marker of plasmacytoid DCs, the major producers of type I IFNs, was downregulated in *Cd38^-/-^* relative to WT spleen cells. Likewise, T cell markers such as *Cd3d*, and *Cd3g* were downregulated, whereas *Ms4a1*, which encodes the B cell marker CD20, and *Cr2*, which encodes CD21, also known as complement receptor type 2, were upregulated (**Supplementary Table S16**). Expression of CD21 is decreased on B lymphocytes of SLE patients, and on B cells from MRL/lpr lupus mice (94). All KEGG pathways showed a higher percentage of downregulated genes in *Cd38^-/-^* relative to WT (**Supplementary Table S17**). The metabolic pathways such as pentose and glucoronate interconversions, and drug metabolism-cytochrome P450, were also downregulated in *Cd38^-/-^* vs WT. These pathways involved a number of UDP-ribosyl transferases isoenzymes, and, to a lesser extent, glutathione transferases, which have direct antioxidant activity. In summary, the major transcriptional differences between *Cd38^-/-^* spleen cells and WT spleen cells were observed 4 weeks after the cGVHD induction, with a strong down-regulation of genes involved in inflammatory, and immune response related pathways, as well in key metabolic pathways.

**Figure 9:**
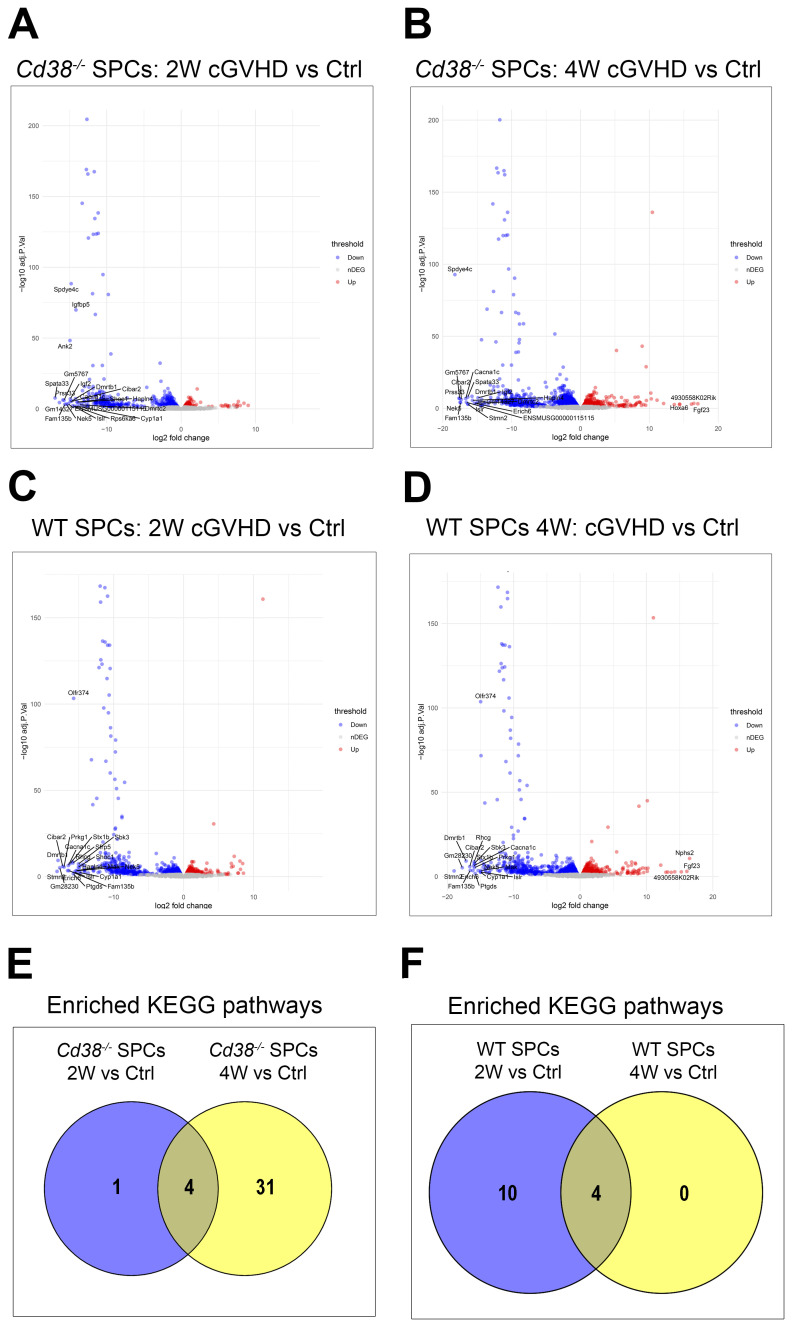
**(A)** Volcano plot of the DEGs upregulated (red circles), and downregulated (blue circles) in *Cd38^-/-^* spleen cells 2 weeks after the bm12 cell transfer vs Control spleen cells from healthy untreated mice. **(B)** Volcano plot of the DEGs upregulated (red circles), and downregulated (blue circles) in *Cd38^-/-^* spleen cells 4 weeks after the bm12 cell transfer vs Control spleen cells from healthy untreated mice. **(C)** Volcano plots of the DEGs upregulated (red circles), and downregulated (blue circles) in WT spleen cells 2 weeks after the bm12 cell transfer vs Control spleen cells from healthy untreated mice. **(D)** Volcano plots of the DEGs upregulated (red circles), and downregulated (blue circles) in WT spleen cells 4 weeks after the bm12 cell transfer vs Control spleen cells from healthy untreated mice. **(E)** Venn diagram showing the number of shared and unique enriched KEGGs for the indicated comparatives. **(F)** Venn diagram showing the number of shared and unique enriched KEGGs for the indicated comparatives.

Second, we asked whether there were differences in the kinetics of the transcriptional activity elicited in *Cd38^-/-^* spleen cells vs WT spleen cells along the course of the disease. In *Cd38^-/-^* spleen cells, 1439 DEGs were detected 2 weeks after the adoptive cell transfer of bm12 cells into *Cd38^-/-^* mice relative to control spleen cells from non-treated *Cd38^-/-^* mice (**Figure 9A**, **Supplementary Table S18**). The number of DEGs increased to 3708 4 weeks after the adoptive transfer relative to healthy non-treated control *Cd38^-/-^* spleen cells (**Figure 9B**, **Supplementary Table S19**). In WT spleen cells, 3622 DEGs were detected at 2 weeks (**Figure 9C**, **Supplementary Table S20**), and 4769 DEGs at 4 weeks of the adoptive transfer of bm12 cells into WT mice, relative to control spleen cells from non-treated WT mice (**Figure 9D**, **Supplementary Table S21**).

5 KEGG pathways were enriched in *Cd38^-/-^* spleen cells 2 weeks after the adoptive transfer relative to control cells (**Supplementary Table S22**). The number of enriched KEGG pathways increased up to 36 at 4 weeks (**Supplementary Table S23**). Venn diagrams showed that 4 pathways were shared by these comparatives, and 31 pathways showed altered transcriptional activity exclusively at 4 weeks of the adoptive transfer (**Figure 9E**). 14 KEGG pathways were significantly enriched in WT spleen cells at 2 weeks after the allogeneic challenge relative to controls (**Supplementary Table S24**), while only 4 KEGG pathways were significantly enriched in WT spleen cells at 4 weeks relative to controls (**Supplementary Table S25**). These 4 pathways were shared with the 2-week comparative (**Figure 9F**).

**Figure 10:**
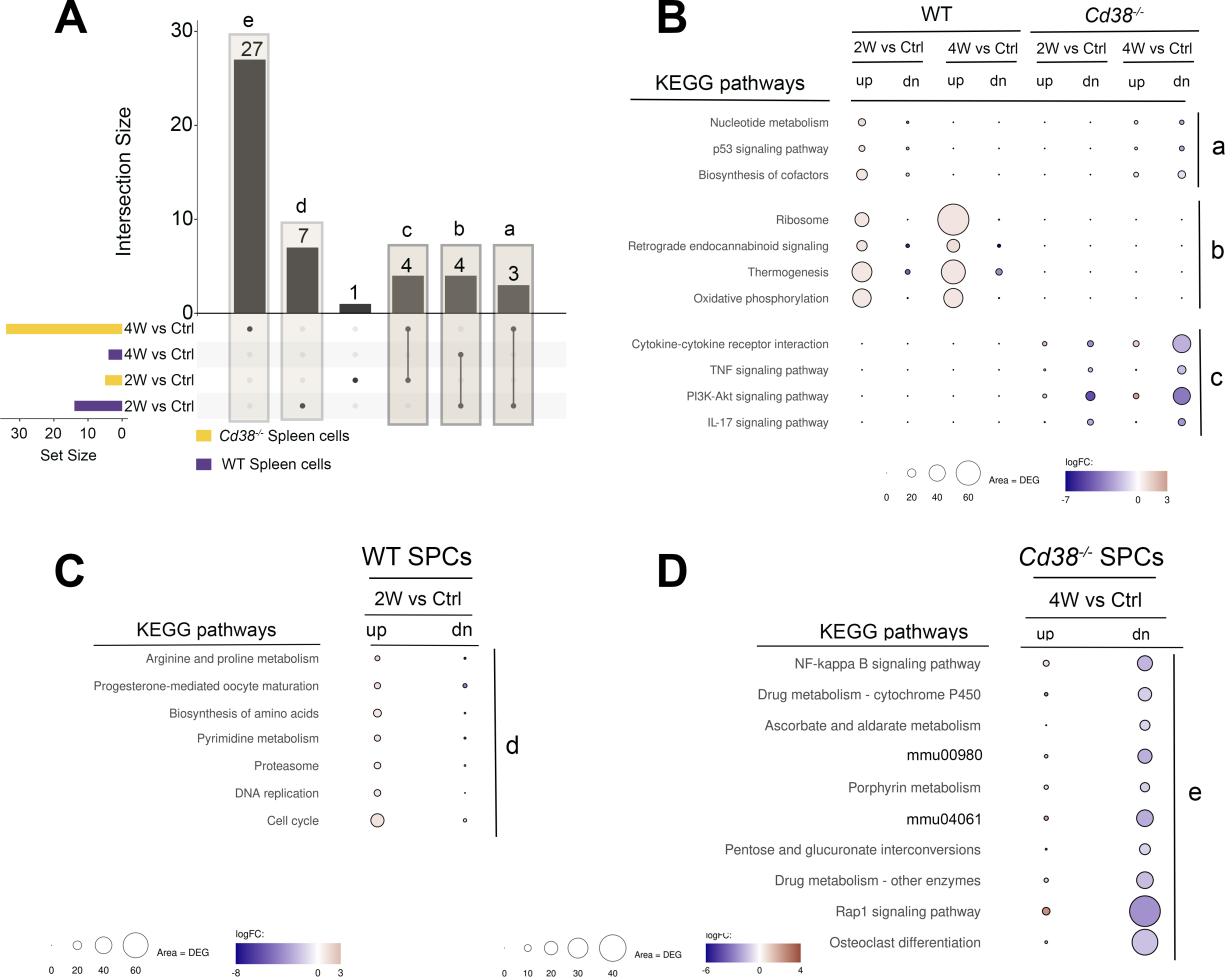
**(A)** The enriched KEGG pathways of these 4-time series or sets of spleen cells comparatives were matched and visualized using UpSet plots. There were not pathways shared by the 4 sets. Five different set intersections are highlighted, a, b, c, d, and e. **(B)** Representation of the KEGG pathways corresponding to the a, b, and c intersections. The intersection a, corresponds to the 3 pathways shared between WT 2-week vs Control and *Cd38^-/-^* 4-week vs Control sets. The intersection b, corresponds to the 4 pathways shared by the 2 WT sets, and the intersection c corresponds to the 4 pathways shared by the 2 *Cd38^-/-^* sets. **(C)** KEGG pathways corresponding to the intersection d, which are exclusively enriched in the WT 2-week vs Control comparative. **(D)** The 10-top out of 27 KEGG pathways exclusively enriched in the *Cd38^-/-^* 4-week vs Control comparative. The mean log_2_FC of the upregulated DEGs (red circles) versus the mean log_2_FC of the downregulated DEGs (blue circles) per pathway were represented graphically. In this representation, the size of the circles was indicative of the number of upregulated or downregulated DEGs, and the intensity of the colors indicate the differences in log_2_FC.

When the enriched KEGG pathways of these 4-time series were matched using UpSet plots, there were not pathways shared by the 4 comparatives or sets (**Figure 10A**). Five different set intersections are highlighted, a, b, c, d, and e. The intersection a, corresponded to the 5 pathways shared between WT 2-week vs Control and *Cd38^-/-^* 4-week vs Control sets (**Figure 10B**, upper part). The enriched pathways in this intersection, nucleotide metabolism, p53 signaling pathway, and biosynthesis of cofactors, showed significant gene upregulation in WT spleen cells at 2 weeks and not at 4 weeks, while in *Cd38^-/-^* spleen cells a relatively lower number of upregulated genes was only observed at 4 weeks of the allogeneic challenge. Moreover, in *Cd38^-/-^* spleen cells, the number of downregulated genes was higher than the number of upregulated genes, further suggesting a delayed and defective activation of these pathways relative to WT.

The intersection b, corresponded to the 4 pathways shared by WT 2-week vs Control and WT 4-week vs Control sets, but not with any of the *Cd38^-/-^* sets (**Figure 10B**, middle). The 4 enriched pathways were ribosome, retrograde endocannabinoid signaling, thermogenesis, and oxidative phosphorylation, and showed larger numbers of upregulated genes than downregulated genes at the two sets, in particular at 4-week vs Control set, which was indicative of a long-lasting activation of these pathways in WT spleen cells.

The intersection c corresponded to the 4 pathways shared by the two *Cd38^-/-^* sets, which included cytokine cytokine-receptor interaction, and the TNF, PI3K-Akt, and IL-17 signaling pathways (**Figure 10B**, bottom). Closer examination of the DEGs associated with these terms showed that there were higher numbers of downregulated genes than upregulated genes, in particular at 4 weeks after the initiation of the allogeneic challenge, phenomenon that was not observed in WT spleen cells.

The intersection d corresponded to the 7 KEGG pathways, which were only enriched in the WT 2-week vs control set (**Figure 10A**). As shown in **Figure 10C**, this intersection included pathways involved in cell proliferation such as cell cycle, DNA replication, pyrimidine metabolism, and nucleotide metabolism, and pathways involved in protein synthesis and degradation such as biosynthesis of amino acids and proteasome. Regarding the p53 signaling pathway, there was not increased expression of the cyclin-dependent kinase inhibitors *Cdkn1a*, and *Cdkn2a*, which are the inhibitors most commonly expressed by senescent cells (**Supplementary Table S24**).

The intersection e corresponded to the 27 enriched KEGG pathways exclusive to the *Cd38^-/-^* 4-week vs control set (**Figure 10A**). The top-10 pathways are shown in **Figure 10D**. All these pathways comprised a larger number of downregulated genes than upregulated genes, which may be indicative of inhibition of these pathways rather than activation.

Overall, the time series results showed long-lasting downregulated expression of key signaling pathways in cGVHD *Cd38^-/-^* spleen cells relative to control healthy spleen cells from non-treated mice, while in cGVHD WT spleen cells there was an initial increase in transcriptomic activity related with proliferation and a long-lasting transcriptional activity in energy-related metabolic pathways, including oxidative phosphorylation and thermogenesis.

## 4 Discussion

In this study RNA sequencing was used to analyze the transcriptome of PECs and spleen cells from *Cd38^-/-^* and WT B6 mice at 2 and 4 weeks of the induction of the cGVHD lupus-like disease, and of PECs and spleen cells from healthy untreated WT and *Cd38^-/-^* mice used as controls. Comparative analysis of these transcriptomes identified a number of pathways associated with the distinct immune and inflammatory response elicited in *Cd38^-/-^* vs WT mice by the transfer of allogeneic bm12 cells, which may be indicative of the transcriptomic differences between the severe lupus disease developed in WT mice versus the milder lupus disease developed in *Cd38^-/-^* mice (18). Another major finding is that the massive transcriptomic reprogramming induced by the allogeneic challenge involves genes related with mitochondria metabolism (oxidative phosphorylation, thermogenesis), with regulated cell death (autophagy, pyroptosis, apoptosis), and with inflammatory reactions elicited by mitochondrial DAMPs (cGAS-STING signaling, inflammasome signaling, senescence), which are indicative of mitochondrial dysfunction (95).

Moreover, we have identified in PECs a distinct transcriptomic profile of purinergic receptors and ectoenzymes involved in the metabolism of the purines in the extracellular space, which were differentially expressed in *Cd38^-/-^* PECs and spleen cells relative to their WT counterparts upon cGVHD induction. Extracellular ATP may function as a DAMP upon binding to cognate receptors expressed on myeloid cells, such as purinergic receptors P2RY2 and P2RX7 (96, 97). The induction of *P2ry2* expression upon bm12 CD4^+^ T cell transfer in *Cd38^-/-^* PECs was significantly lower than in WT PECs. The purinergic receptor P2Y2 binds ATP and stimulates chemotaxis of various cells including macrophages, neutrophils, eosinophils, and others, and global P2Y2 knockout mice exhibit impaired myeloid cell chemotaxis and are protected in various models of acute inflammation (75, 96). Likewise, *P2ry12* increased expression was short-lived in *Cd38^-/-^* PECs, while sustained in WT PECs. Its activation induces rapid chemotaxis in response to tissue injury (75), and plays a critical role in the regulation of Th17 differentiation and EAE pathogenesis (98). Within the purinergic receptor family, *P2ry6* and *P2ry13* are the most strongly upregulated genes in WT PECs, and to a lesser extent in *Cd38^-/-^* PECS upon cGVHD induction. P2RY13 is highly expressed in spleen, lymph nodes and bone marrow, is structurally related to P2RY12 sharing a high affinity for ADP (99), and P2RY13-mediated signaling protects against metabolic dysfunctions associated with obesity (100). P2RY6 protein responds to extracellular UPD, which acts as an “eat-me” signal for P2Y6R, and performs its response through phospholipase C signaling pathway triggering IP3, which then induces a steady accumulation of intracellular Ca^2+^ levels (101), and may also participate in the regulation of inflammation (102–105). On the other hand, the adoptive-transfer-dependent upregulation of *P2rx4*, and *P2rx7* expression seemed to be more stable and interconnected with various signaling pathways such as Ca^2+^-mediated signals (**Figure 3B**), and NLRP3 activation. On the same line, P2RX1 and P2RX4 localized to the immune synapse close to mitochondria, and trigger a localized Ca^2+^ influx that stimulates OXPHOS and propagates TCR-induced signaling, culminating in cytokine secretion and T cell proliferation (106). In our study, we have shown that in PECs, the differential expression of 11 purinergic receptors genes in response to an allogeneic challenge, may constitute a transcriptomic signature that discriminate between severe lupus and mild lupus disease. Moreover, most of these receptors seem to be downregulated in cGVHD spleen cells relative to control cells, highlighting the importance of the purinergic receptors signaling in the peritoneal cavity, where a significant proportion of inflammatory Ly6C^hi^ monocytes are present in cGVHD WT mice relative to cGVHD *Cd38^-/-^* mice.

P2RX7 is a bifunctional receptor for extracellular ATP that, depending on the level of activation, forms a cation-selective channel or a large conductance nonselective pore. P2X7 has a strong pro-apoptotic activity but can also support growth (107). Extracellular NAD^+^ induces the ATP-independent activation of P2X7 in murine T cells via ADP-ribosylation of P2X7 catalyzed by ART2.2 (108). P2X7R activation by ART2.2 triggers calcium flux, phosphatidylserine exposure, L-selectin (CD62L) shedding, loss of the mitochondrial membrane potential and pore formation, resulting in cell death (108). In the cGVHD lupus model *Art2b*, which encodes ART2.2, is upregulated in WT PECs, and to a lesser extent in *Cd38^-/-^* PECs. *Art2a* encodes ART2.1, which in C57BL/6 mice is considered a pseudogene, because a polymorphism creates a premature stop codon at position 161 that produces a truncated protein that is not functional (109). P2RX7 also has a substantial role in the expansion, metabolic reprogramming and effector functions of CD8^+^ memory T cells (110).

*Nlrp3* encodes NLRP3, which forms inflammasome complexes to activate Capase-1, which in turn cleaves pro-IL1β and pro-IL-18, allowing their secretion as mature IL-1β and IL-18 and the consequent induction of inflammation. *Il1b* and the other two components of the inflammasome, *Pycard*, and *Casp1* were also overexpressed in WT PECs upon the allogeneic challenge. Moreover, *P2rx7*, *Trpm2*, and *Tlr4* were upregulated, which are genes encoding potential activators of the NLRP3-inflammasome complex (86, 87). On the basis of studies in mouse macrophages, activation of the NLRP3 inflammasome is thought to require two signals. The first signal can be provided by TLR stimulation and triggers the synthesis of pro-IL-1β and NLRP3. The second signal can be mediated by stimulation of P2RX7 by ATP resulting in K^+^ efflux, Ca^2+^ influx, and other signals that activate the inflammasome (75). ATP released from dying cancer cells activate P2X7 receptors on DCs leading to activation of the NLRP3 inflammasome, IL-1β release and priming of IFN-γ-producing tumor antigen-specific CD8^+^ T cells (97). Therefore, inflammasome activation can establish a link between the innate (inflammatory) and the acquired (cognate) immune responses, because IL-1β produced by DCs is required for the priming of T cells (97). Whether this mechanism is operative in the cGVHD lupus model, where donor bm12 CD4^+^ T cells activated by host MHC II provide cognate help for host B cells to initiate lupus (17, 22), requires further investigation.

The cGAS-STING signaling pathway is known to trigger type I IFN responses after free cytosolic DNA binds to the cytoplasmic dsDNA sensor cGAS leading to the production of a second messenger cGAMP, which binds to and activates STING, resulting in downstream TBK1 activation, and increased type I IFN production (111). In this study, WT PECs showed a strong and persistent increase in the gene expression of *Cgas*, and a number of downstream ISGs associated with the *in vivo* type I IFN response, while in *Cd38^-/-^* PECs increased expression of these genes peaked at 2 weeks, with a strong decline at 4 weeks after the allogeneic challenge. Any DNA released or leaked from the nucleus and mitochondria into the cytoplasm might trigger the cGAS-STING pathway (112). Alternatively, Ifi-204 protein, which is another cytosolic DNA sensor, which its gene is strongly upregulated in WT PECs after cGVHD induction, may mediate type I IFN induction via recruiting STING (90, 91). IFI16, the human orthologous of Ifi-204, is a SLE autoantigen that bind neutrophil extracellular traps and elicits anti-IFI16 auto-antibodies (113).

In contrast, gene expression of *Tlr9*, another DNA sensor that stimulates type I IFN, was downregulated in both WT and *Cd38^-/-^* PECs. Recently it has been reported that CD38 can inhibit extracellular cGAMP activity through its direct binding (114). The authors speculated that given the fact that CD38 exists in two opposite configurations on the cell membrane, facing either the extracellular or the intracellular environment (115), part of the decoy activity of CD38 toward cGAMP may occur in the intracellular space (114), where the cGAMP concentration is in the micromolar range (116). In this scenario, in cells that highly express CD38 such as B cells, intracellular CD38 may regulate STING activation.

In the bm12-cGVHD lupus model, apoptosis is one of the most significant enriched KEGG pathways shown in WT PECs at both time points, and in *Cd38^-/-^* PECs 2 weeks after the allogeneic challenge. Accumulated apoptotic-derived molecules, including self-DNA, may serve as autoantigens, and drive autoantibody production. Alternatively, another mode of cell death, ferroptosis, which was also an enriched KEG pathway in WT PECs, could contribute to the release of autoantigens, including dsDNA, providing additional stimuli to autoreactive B cells and pDCs to produce autoantibodies and type I IFNs, respectively. In this sense, STING regulates CD4^+^ T cell and B cell responses in the cGVHD lupus model (117). Given that the transferred bm12-CD4^+^ T cells were STING sufficient, these data further support the role for Ag-presenting, cell-intrinsic STING in the induction of adaptive immune responses to dying cell-derived antigens (117). STING mediates lupus via the activation of conventional dendritic cell maturation and plasmacytoid dendritic cell differentiation in the Fcgr2b-deficient lupus mice (118), and increased cGAS expression and cGAMP in a proportion of SLE patients indicated that the cGAS/cGAMP pathway may play a role in disease expression (119). Moreover, apoptosis-derived membrane vesicles drive the cGAS-STING pathway and enhance type I IFN production in SLE (120).

Regarding the mechanisms that regulate the SASP phenotype in cellular senescence, NAD^+^ metabolism supports metabolic changes and signaling that leads to the activation of NF-κB signaling and the expression of genes associated with high pro-inflammatory SASP by upregulating NAMPT expression (81). In our study, *Nampt* gene expression is upregulated in the same samples were cellular senescence and NOD-like receptor signaling pathways are enriched. NAMPT can exert a number of functions as an extracellular cytokine, including activation of the NLRP3 inflammasome (121) and premature senescence in endothelial cells (122). Whether any of these mechanisms are activated in the cGVHD lupus model requires further investigation. Genetic deletion of CD38 rescued age-related changes in gene expression in ovaries of middle-aged mice, particularly genes related to the senescence pathway (123). Cellular senescence in immune cells, also known as immunosenescence (124, 125), often occurs in the setting of chronic inflammation. In this sense, it has been shown that CD38 is a transcriptional target of the nuclear receptor liver X receptor (LXR), which is activated by derivatives of cholesterol metabolism (126), and recently published data describes a mechanism whereby cholesterol promotes macrophage senescence by NAD^+^ depletion via LXR/CD38 signaling (127). Moreover, inflammatory mediators and LXRs synergistically induced the expression of the multifunctional protein CD38 (128).

Our data on enrichment in metabolic pathways related with energy expenditure suggests that in *Cd38^-/-^* spleen cells the adoptive transfer of bm12 cells into the peritoneal cavity causes a down-regulation of genes involved in the regulation of the glycolytic pathway without an apparent increase in the genes involved in oxidative phosphorylation. In contrast, in WT spleen cells there was a strong metabolic reprogramming 2 weeks upon the adoptive transfer of bm12 cells relative to that in WT spleen cells from healthy untreated mice, with increased expression of genes involved in the generation of ATP via oxidative phosphorylation.

The kinetics of this metabolic reprogramming differs in *Cd38^-/-^* PECs. Thus, the time series results show strong upregulation of genes related with oxidative phosphorylation in *Cd38^-/-^* PECs 2 weeks after the adoptive transfer of bm12 cells, which is no longer maintained at 4 weeks. In contrast, in WT PECs upregulation of oxidative phosphorylation-related genes is more robust and sustained in time, with many transcripts associated with the conversion of NADH to NAD^+^ and ADP to ATP. To note is that upregulation of genes related with glycolysis/gluconeogenesis and fatty acid oxidation is exclusively found in WT PECs at 2 weeks, which strongly suggests that alternative sources of energy are used by WT PECs upon allogeneic challenge.

We have previously shown in cGVHD lupus model the failure of host *Cd38^-/-^* B cells to fully activate and expand CD38-sufficient donor bm12 T cells (18). In this setting the cognate interaction occurs between CD4^+^ T cells that are CD38 sufficient, and B cells, which are CD38 negative, and unable of providing a strong signal to maintain and expand the donor T cells, and recipient B cells. It is likely that fully expansion of these cells, as it occurs in WT cGVHD mice, would require an initial fast source of energy provided by aerobic glycolysis and fatty acid oxidation, followed by a prolonged energy expenditure, which could be provided mainly via oxidative phosphorylation. In this sense, Yin et al. (129) reported that glycolysis and mitochondrial oxidative metabolism are elevated in cells from SLE patients as well as in mouse models of disease, including the cGVHD model. Moreover, inhibitors of these pathways currently in the clinic—2-deoxy-d-glucose (2DG) and metformin—normalize T cell metabolism and decrease markers of SLE in animal models as well as in cells from SLE patients (93).

On the other hand, there is now evidence that many aspects of metabolic regulation are cell-specific (93, 130), and in our study some of the results obtained in the time series comparatives may reflect the expansion of monocytes/macrophages in both the peritoneal cavity (this study), and in the spleen in response to the allogeneic challenge (18). Alternatively, metabolic reprogramming may occur as consequence of inflammatory and host defense mechanisms (131). Thus, in our system TLR and NOD-like receptor agonists and cytokines can influence the balance of anabolic to catabolic metabolism leading to mitochondrial biogenesis and increased oxidative phosphorylation, which may be critical for the long-term survival of immune cells and the persistence vs resolution of the autoimmune and inflammatory process (131).

## 5 Conclusions

**Figure 11:**
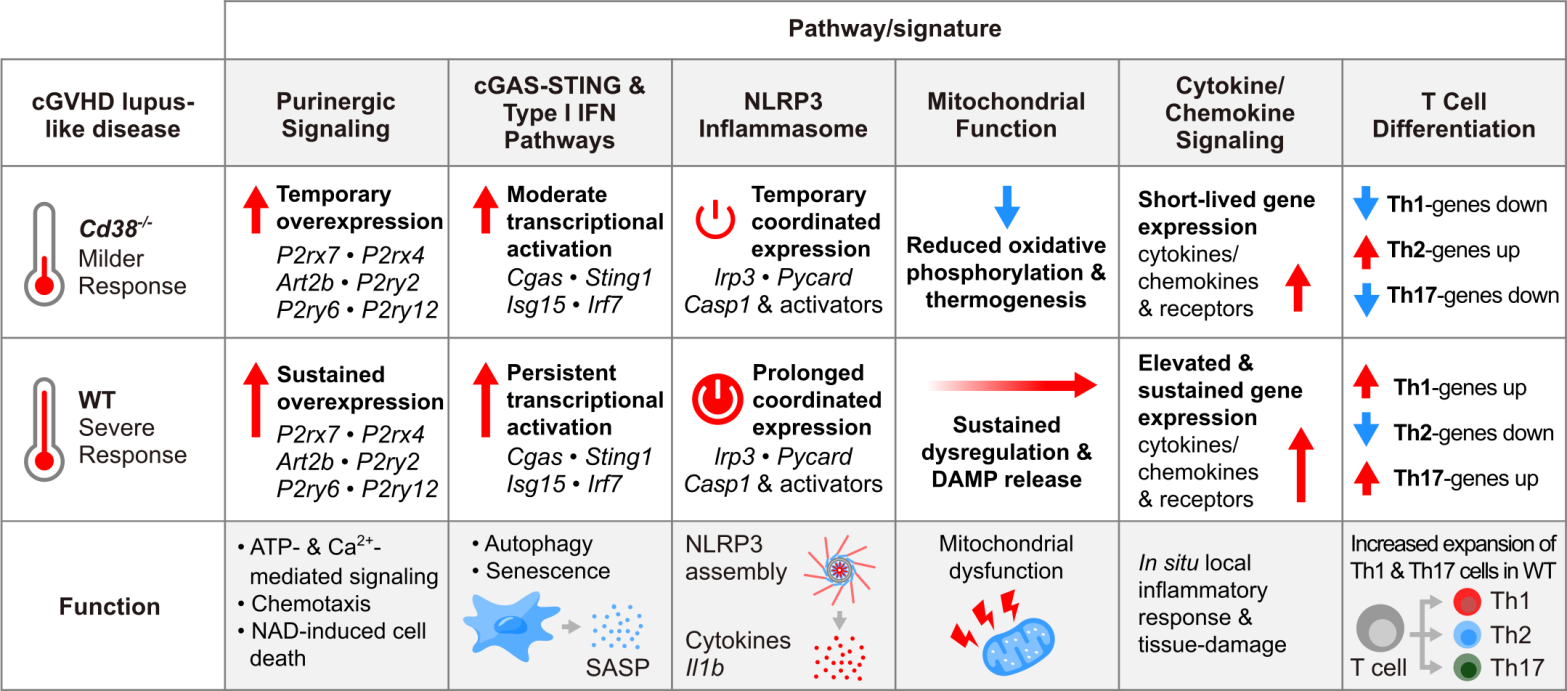
Summary of the major findings in this study, and associated functions. Design: NorArte Visual Science (https//norarte.es).

In this study RNA sequencing was used to analyze the transcriptome of PECs and spleen cells from *Cd38^-/-^* and WT B6 mice at 2 and 4 weeks after the induction of the cGVHD lupus-like disease, and of PECs and spleen cells from healthy untreated WT and *Cd38^-/-^* mice used as controls (see **Figure 11** for a summary of the major findings). Comparative analysis of these transcriptomes identified a number of pathways associated with the distinct immune and inflammatory response elicited in *Cd38^-/-^* vs WT mice by the transfer of allogeneic bm12 cells. For most of them, these transcriptomic profiles were tissue-specific, and the transcriptional activity was more intense and long-lasting in WT than in *Cd38^-/-^* samples. Thus, in PECs we have identified a distinct transcriptomic profile of purinergic receptors and purinergic ectoenzymes involved in the purines metabolism in the extracellular space. The transcriptomic expression profile of these receptors and ectoenzymes seemed to be coordinately expressed with genes of cGAS-STING signaling pathway and a number of interferon stimulated genes, two hallmarks in the lupus pathology. A second purinergic receptor transcriptomic profile showed an apparent coordinated gene expression of the components of the NLRP3 inflammasome with its potential activators. These processes were transcriptionally less active in *Cd38^-/-^* PECs than in WT PECs upon the allogeneic challenge, and to our knowledge, it has not been previously reported in the literature. In this study we have also shown evidence of a distinct enrichment in pathways signatures that define processes such as Ca^2+^ ion homeostasis, cell division, phagosome, autophagy, senescence, cytokine/cytokine receptor interactions, Th17 and Th1/Th2 cell differentiation in *Cd38^-/-^* vs WT samples, which reflected the milder inflammatory and autoimmune response elicited in *Cd38^-/-^* mice relative to WT counterparts in response to the allogeneic challenge (**Figure 11**). Last, we have shown a strong metabolic reprogramming toward oxidative phosphorylation in spleen cells and PECs from WT cGVHD mice, which it may reflect an increased cellular demand for oxygen consumption, in contrast to spleen cells, and PECs from *Cd38^-/-^* cGVHD mice, which showed a short-lived metabolic effect at transcriptomic level. Although we have not done functional experiments to corroborate these findings, our data are in agreement with other authors that have suggested that chronic T cell activation by autoantigens (as it occurs in SLE) is supported by oxidative phosphorylation, whereas aerobic glycolysis supports acute activation induced by foreign antigens, or *in vitro* supra-physiological TCR stimulation (93).

## Data Availability

The data presented in the study are available at SRA, BioProject PRJNA1118233.
