# Supplementary material for: CD38 deficiency leads to a defective short-lived transcriptomic response to chronic graft-versus-host disease induction, involving purinergic signaling-related genes and distinct transcriptomic signatures associated with lupus
Source: Front Immunol. 2025 Feb 10;16:1441981. doi: 10.3389/fimmu.2025.1441981 (PMC11847871; doi:10.3389/fimmu.2025.1441981)
Supplement: Supplementary file 1 [file DataSheet1.zip › Supplemental Fig_1441981_Dic 24/Figure S1 with Figure legend (1).pdf]

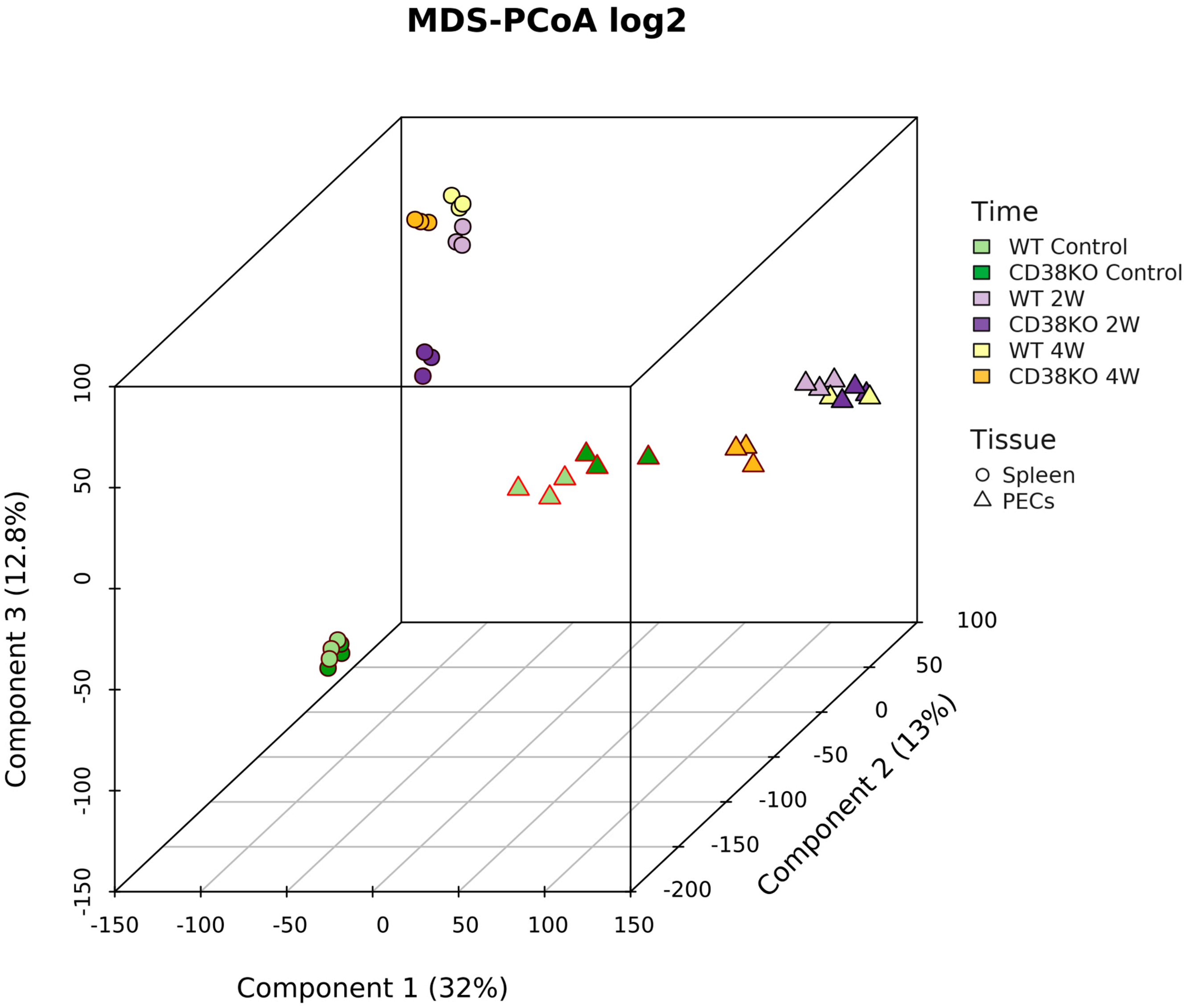

Figure S1. Three dimensional representation of Principal Corresondence Analysis (PCoA) also known as Metric Multidimensional Scaling (MDS) on the Euclidian distnaces among the samples used in this study. As shown in this representation biological replicates clustered at very short distance, while spleen samples were very well separated from PECs samples as expected.
