# Supplementary material for: CD38 deficiency leads to a defective short-lived transcriptomic response to chronic graft-versus-host disease induction, involving purinergic signaling-related genes and distinct transcriptomic signatures associated with lupus
Source: Front Immunol. 2025 Feb 10;16:1441981. doi: 10.3389/fimmu.2025.1441981 (PMC11847871; doi:10.3389/fimmu.2025.1441981)
Supplement: Supplementary file 1 [file DataSheet1.zip › Supplemental Fig_1441981_Dic 24/FIGURE S2_with legend.pdf]

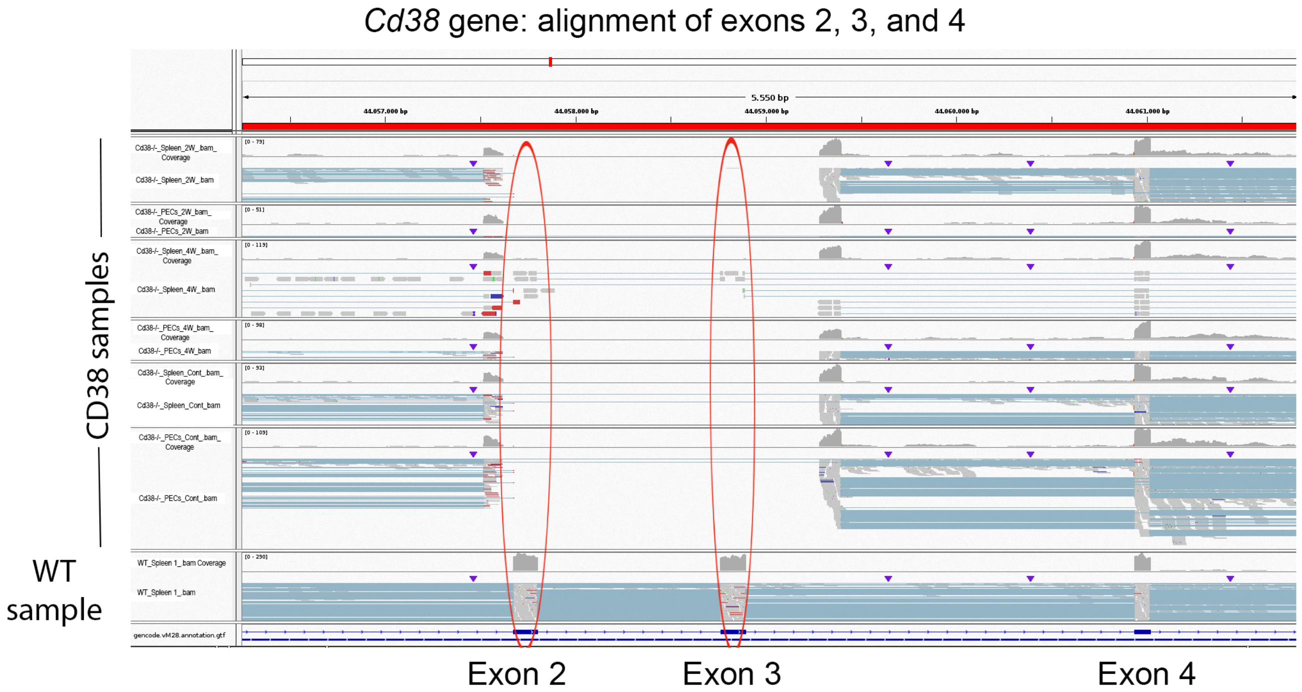

Figure S2. Visualization of deleted *Cd38* gene exons 2 and 3 in *Cd38*$^{-/-}$ samples vs a WT sample.The image was generated from BAM files post-alignment of the samples using the IGV visualizer. The region of interest depicts the deletion of exons 2 and 3 (highlighted in red, while exon 4 is conserved.
