# Supplementary material for: CD38 deficiency leads to a defective short-lived transcriptomic response to chronic graft-versus-host disease induction, involving purinergic signaling-related genes and distinct transcriptomic signatures associated with lupus
Source: Front Immunol. 2025 Feb 10;16:1441981. doi: 10.3389/fimmu.2025.1441981 (PMC11847871; doi:10.3389/fimmu.2025.1441981)
Supplement: Supplementary file 1 [file DataSheet1.zip › Supplemental Fig_1441981_Dic 24/Figure_S3_with Figure legend.pdf]

A

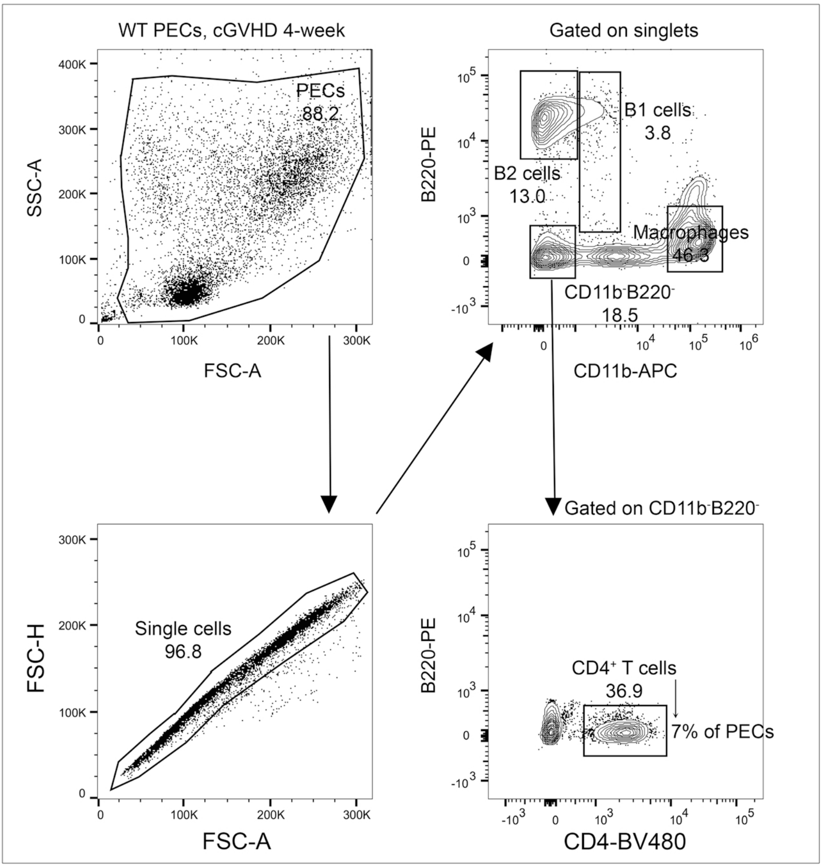

B

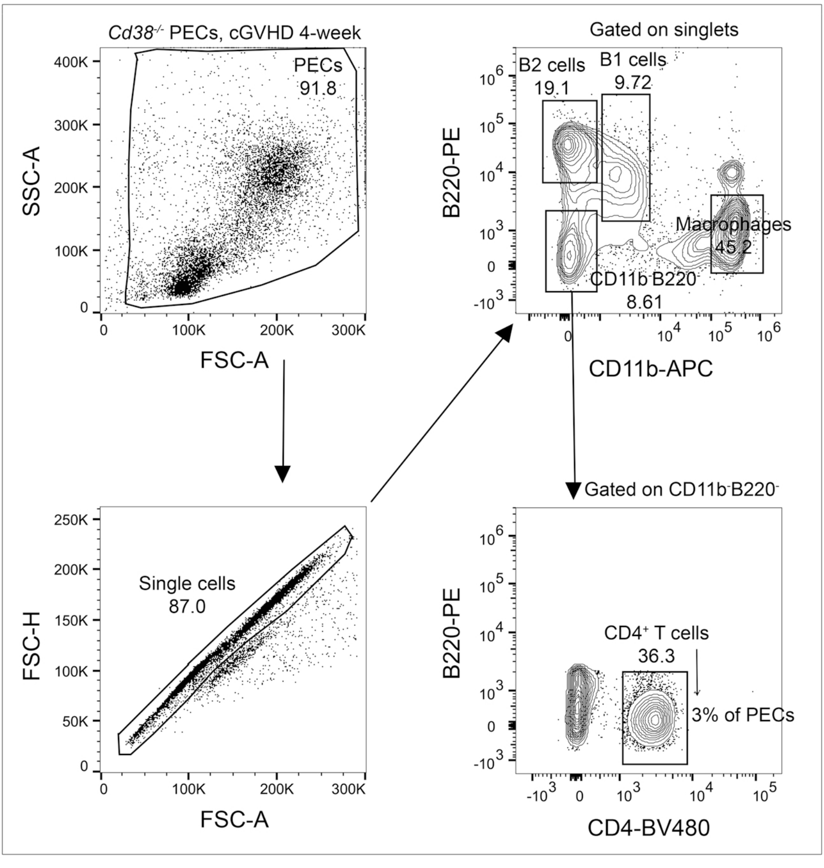

Figure S3. Gating strategy to identify B1 cells, B2 cells, macrophages, and CD4+ T cells in WT PECs (A), or in *Cd38*$^{-/-}$ PECs (B), 4 weeks after cGVHD induction. B1cells: B220^{int}CD11b^{lo}; B2 cells: B220^{hi}CD11b^{-}; Macrophages: B220^{-}CD11b^{hi}; CD4^{+} T cells: B220^{-}CD11b^{-}CD4^{+}
