# Supplementary material for: CD38 deficiency leads to a defective short-lived transcriptomic response to chronic graft-versus-host disease induction, involving purinergic signaling-related genes and distinct transcriptomic signatures associated with lupus
Source: Front Immunol. 2025 Feb 10;16:1441981. doi: 10.3389/fimmu.2025.1441981 (PMC11847871; doi:10.3389/fimmu.2025.1441981)
Supplement: Supplementary file 1 [file DataSheet1.zip › Supplemental Fig_1441981_Dic 24/Figure_S4_with Figure legend.pdf]

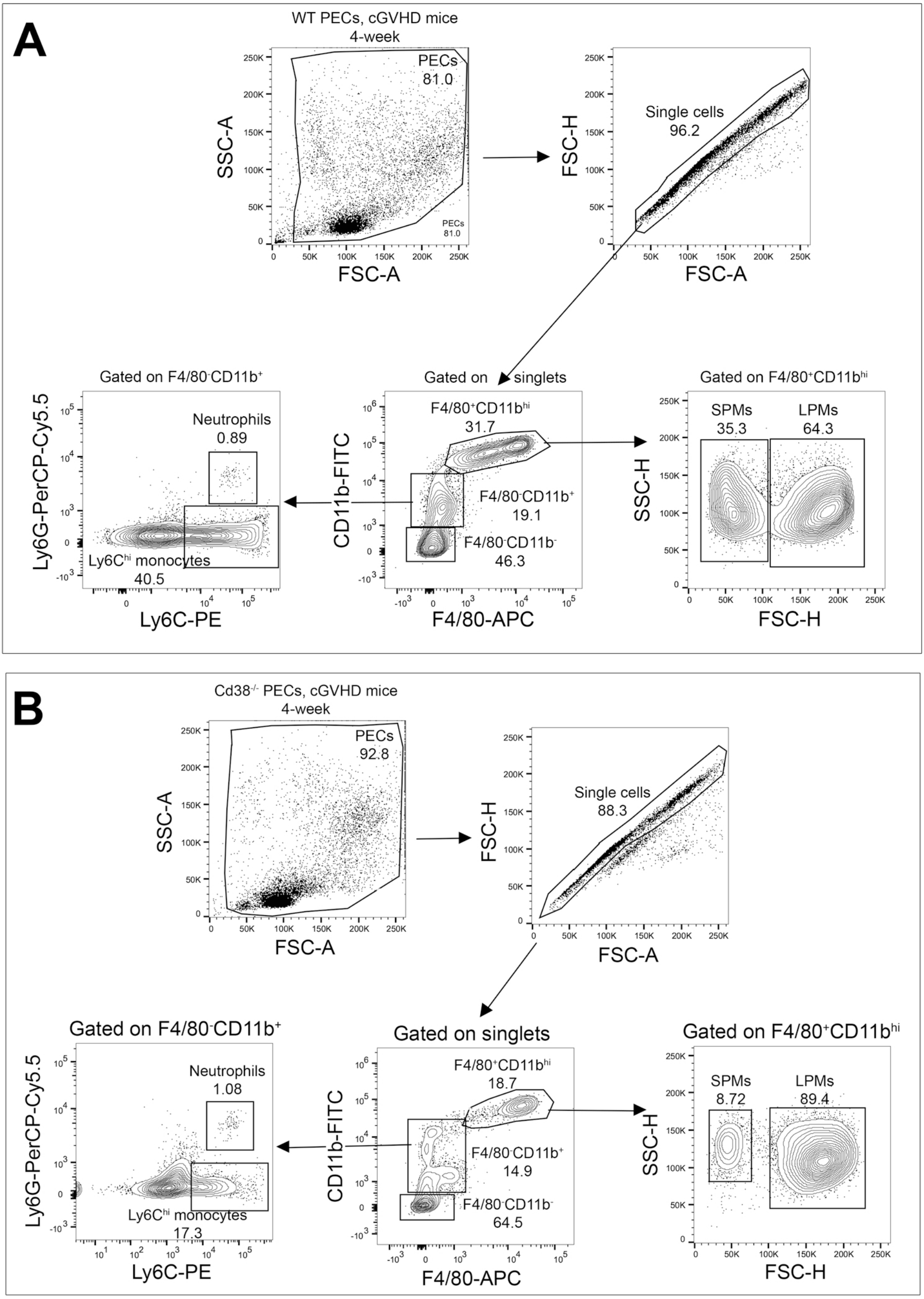


Figure S4. Gating strategy to identify large macrophages (LPMs), small macrophages (SPMs), neutrophils, Ly6C$^{hi}$ monocytes, and lymphoid cells in WT PECs (A), or in *Cd38*$^{-/-}$ PECs (B), 4 weeks after cGVHD induction. LPMs: F4/80$^{+}$CD11b$^{hi}$FSC$^{hi}$; SPMs: F4/80$^{+}$ CD11b$^{hi}$FSC$^{lo}$; Neutrophils: F4/80$^{-}$CD11b$^{+}$Ly6C$^{+}$Ly6G$^{+}$; Ly6C$^{hi}$ monocytes:F4/80$^{-}$CD11b$^{+}$Ly6C$^{hi}$Ly6G$^{-}$; Lymphoid cells: F4/80$^{-}$CD11b$^{-}$
