# Supplementary figures and images for: CD38 deficiency leads to a defective short-lived transcriptomic response to chronic graft-versus-host disease induction, involving purinergic signaling-related genes and distinct transcriptomic signatures associated with lupus
Source: Front Immunol. 2025 Feb 10;16:1441981. doi: 10.3389/fimmu.2025.1441981 (PMC11847871; doi:10.3389/fimmu.2025.1441981)

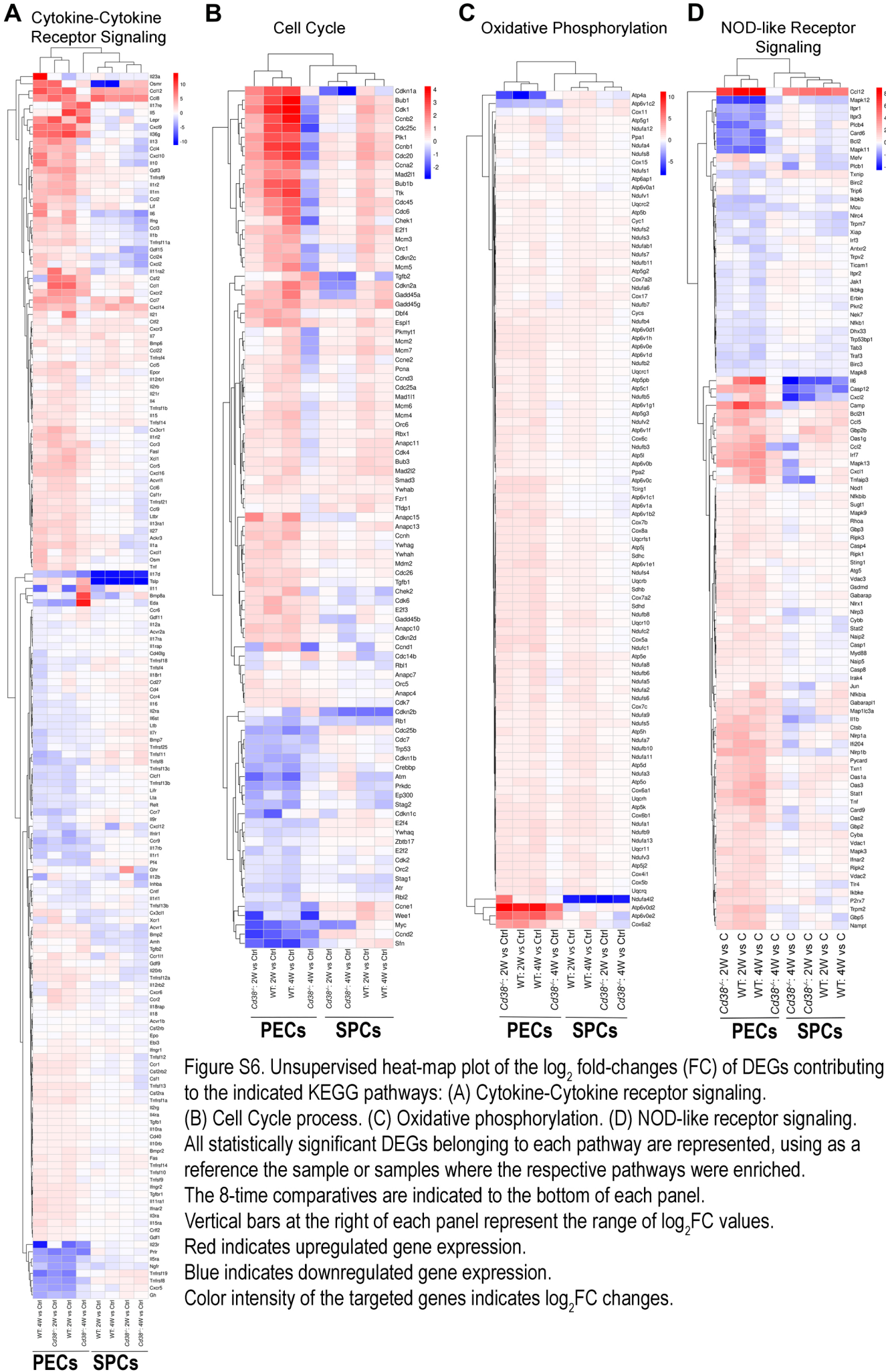

Supplement: Supplementary file 1 [file DataSheet1.zip › Supplemental Fig_1441981_Dic 24/Figure_S6_with Figure legend.pdf]
