# Supplementary material for: CD38 deficiency leads to a defective short-lived transcriptomic response to chronic graft-versus-host disease induction, involving purinergic signaling-related genes and distinct transcriptomic signatures associated with lupus
Source: Front Immunol. 2025 Feb 10;16:1441981. doi: 10.3389/fimmu.2025.1441981 (PMC11847871; doi:10.3389/fimmu.2025.1441981)
Supplement: Supplementary file 2 [file DataSheet2.zip › DEGs_KEGGs_Tables_171124_1441981/Table S1 ___Antibodies Flow Cytometry 290524 noridd.pdf]

TABLE S1, Anti-mouse antibodies for flow cytometry

**Antibodies anti-mouse**	**Dilution**	**Company**
TCR-β-FITC	1:500	BD Biosciences Cat# 553170
CXCR5-biotin	1:350	BD Biosciences Cat# 551960
PD1-APC	1:350	BD Biosciences Cat# 562671
Streptavidin-PE	1:350	BioLegend Cat# 405203
CD4 BV480	1:250	BD Biosciences Cat# 565634
CD4-PercP	1:250	BD Biosciences Cat#561090
CXCR5-PE	1:125	BD Biosciences Cat#561988
CD11b-APC	1:250	BioLegend Cat#101211
Ly6G-PerCP-Cy5.5	1:250	(BioLegend Cat#127615
Ly6C-PE	1:250	BioLegend Cat#128007
CD11b-FITC	1:250	BioLegend Cat#101205
F4/80-APC	1:250	BioLegend Cat#123115
CD38-FITC	1:250	BD Biosciences Cat# 558813
CD38-BB700	1:250	BD Biosciences Cat# 742132
CD5-PerCP	1:250	BD Biosciences Cat# 553025
CD45R/B220-PE	1:350	BD Biosciences Cat# 553089
