# Supplementary material for: CD38 deficiency leads to a defective short-lived transcriptomic response to chronic graft-versus-host disease induction, involving purinergic signaling-related genes and distinct transcriptomic signatures associated with lupus
Source: Front Immunol. 2025 Feb 10;16:1441981. doi: 10.3389/fimmu.2025.1441981 (PMC11847871; doi:10.3389/fimmu.2025.1441981)
Supplement: Supplementary file 2 [file DataSheet2.zip › DEGs_KEGGs_Tables_171124_1441981/Table S2_pdf.pdf]

Supplemental Material
Table S2: Samples

ID Number	ID samples	DESCRIPTION
NGS073-21-1	**AB3**	**Cd38-/-_Spleen 1_2W**
NGS073-21-2	**AB4**	**Cd38-/-_Spleen 2_2W**
NGS073-21-3	**AB6**	**Cd38-/-_Spleen 3_2W**
NGS073-21-4	**AB7**	**Cd38-/-_PECs 1_2W**
NGS073-21-5	**AB54**	**Cd38-/-_PECs 2_2W**
NGS073-21-6	**AB55**	**Cd38-/-_PECs 3_2W**
NGS073-21-7	**AB23**	**Cd38-/-_Spleen 1_4W**
NGS073-21-8	**AB24**	**Cd38-/-_Spleen 2_4W**
NGS073-21-9	**AB25**	**Cd38-/-_Spleen 3_4W**
NGS073-21-10	**AB28**	**Cd38-/-_PECs 1_4W**
NGS073-21-11	**AB59**	**Cd38-/-_PECs 2_4W**
NGS073-21-12	**AB60**	**Cd38-/-_PECs 3_4W**
NGS073-21-13	**AB40**	**Cd38-/-_Spleen 1_Control**
NGS073-21-14	**AB41**	**Cd38-/-_Spleen 2_Control**
NGS073-21-15	**AB42**	**Cd38-/-_Spleen 3_Control**
NGS073-21-16	**AB43**	**Cd38-/-_PECs 1_Control**
NGS073-21-17	**AB44**	**Cd38-/-_PECs 2_Control**
NGS073-21-18	**AB45**	**Cd38-/-_PECs 3_Control**
NGS073-21-19	**AB11**	**WT_Spleen 1_2W**
NGS073-21-20	**AB14**	**WT_Spleen 2_2W**
NGS073-21-21	**AB15**	**WT_Spleen 3_2W**
NGS073-21-22	**AB16**	**WT_PECs 1_2W**
NGS073-21-23	**AB18**	**WT_PECs 2_2W**
NGS073-21-24	**AB19**	**WT_PECs 3_2W**
NGS073-21-25	**AB29**	**WT_Spleen 1_4W**
NGS073-21-26	**AB30**	**WT_Spleen 2_4W**
NGS073-21-27	**AB31**	**WT_Spleen 3_4W**
NGS073-21-28	**AB35**	**WT_PECs 1_4W**
NGS073-21-29	**AB62**	**WT_PECs 2_4W**
NGS073-21-30	**AB47**	**WT_Spleen 1_Control**
NGS073-21-31	**AB48**	**WT_Spleen 2_Control**
NGS073-21-32	**AB49**	**WT_Spleen 3_Control**
NGS073-21-33	**AB50**	**WT_PECs 1_Control**
NGS073-21-34	**AB51**	**WT_PECs 2_Control**
NGS073-21-35	**AB52**	**WT_PECs 3_Control**
