# Supplementary material for: CD38 deficiency leads to a defective short-lived transcriptomic response to chronic graft-versus-host disease induction, involving purinergic signaling-related genes and distinct transcriptomic signatures associated with lupus
Source: Front Immunol. 2025 Feb 10;16:1441981. doi: 10.3389/fimmu.2025.1441981 (PMC11847871; doi:10.3389/fimmu.2025.1441981)
Supplement: Supplementary file 1 [file Figure_S5_with Figure legend.pdf › Supplemental Fig_1441981_Dic 24/Figure_S5_with Figure legend.pdf]

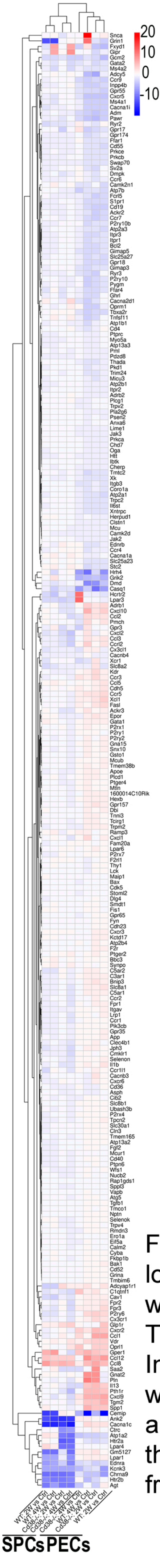

Figure S5. Unsupervised hierarchical heat-map plot of the log_2FC of the relative expression of the genes associated with the GO term, Ca^{2+} ion homeostasis (GO:0055074). This GO term was enriched in several comparatives. In this figure, all statistically significant DEGs associated with this GO term are represented. The eight comparatives are indicated at the bottom. Top dendograms indicate that there was a perfect clustering of PECs samples separated from the spleen samples.
